# Supplementary material for: Chromosome Xq23 is associated with lower atherogenic lipid concentrations and favorable cardiometabolic indices
Source: Nat Commun. 2021 Apr 12;12:2182. doi: 10.1038/s41467-021-22339-1 (PMC8042019; doi:10.1038/s41467-021-22339-1)
Supplement: Supplementary file 1 — Supplementary Information [file 41467_2021_22339_MOESM1_ESM.pdf]

SUPPLEMENTARY INFORMATION

Chromosome Xq23 is associated with lower atherogenic lipid concentrations and favorable cardiometabolic indices

Supplementary Note 1. NHLBI TOPMed program study participants

Atherosclerosis Risk in Communities study (ARIC, 7,991)

TOPMed dbGaP accession#: phs001211, Parent dbGaP accession#: phs000280

ARIC is a large population-based prospective longitudinal cohort study (began 1987) from four U.S. communities: Forsyth County, NC; Jackson, MS; the northwest suburbs of Minneapolis, MN; and Washington County, MD. ARIC was designed to investigate the etiology and natural history of atherosclerosis, its consequences, and related medical care by race, gender, location, and time as previously described.[1] A total of 15,792 participants (55% female and 27% African American) aged 45-64 years were recruited between 1987 and 1989 and received extensive examination, including medical, social and demographic date. The baseline visit was conducted between 1987 and1989, the second visit in 1990-1992, the third visit in 1993-1995, the fourth visit in 1996-1998, the fifth visit in 2011-2013, the sixth visit in 2016-2017, and the seventh visit in 2018-2019. Follow-up is also conducted semi-annually since 2012 by telephone to maintain contact with participants and to assess the health status of the cohort.

The Atherosclerosis Risk in Communities study has been funded in whole or in part with Federal funds from the National Heart, Lung, and Blood Institute, National Institutes of Health, Department of Health and Human Services (contract numbers HHSN268201700001I, HHSN268201700002I, HHSN268201700003I, HHSN268201700004I and HHSN268201700005I). The authors thank the staff and participants of the ARIC study for their important contributions.

Whole genome sequencing (WGS) for the Trans-Omics in Precision Medicine (TOPMed) program was supported by the National Heart, Lung and Blood Institute (NHLBI). WGS for “NHLBI TOPMed: Atherosclerosis Risk in Communities (ARIC)” (phs001211) was performed at the Baylor College of Medicine Human Genome Sequencing Center (HHSN268201500015C and 3U54HG003273-12S2) and the Broad Institute for MIT and Harvard (3R01HL092577-06S1). Centralized read mapping and genotype calling, along with variant quality metrics and filtering were provided by the TOPMed Informatics Research Center (3R01HL-117626-02S1). Phenotype harmonization, data management, sample-identity QC, and general study coordination, were provided by the TOPMed Data Coordinating Center (3R01HL-120393-02S1). We gratefully acknowledge the studies and participants who provided biological samples and data for TOPMed.

The Genome Sequencing Program (GSP) was funded by the National Human Genome Research Institute (NHGRI), the National Heart, Lung, and Blood Institute (NHLBI), and the National Eye Institute (NEI). The GSP Coordinating Center (U24 HG008956) contributed to cross-program scientific initiatives and provided logistical and general study coordination. The Centers for Common Disease Genomics (CCDG) program was supported by NHGRI and NHLBI, and whole genome sequencing was performed at the Baylor College of Medicine Human Genome Sequencing Center (UM1 HG008898 and R01HL059367).

Old Order Amish (Amish, 1,083)

TOPMed dbGaP accession#: phs000956, Parent dbGaP accession#: phs000391

The Amish Complex Disease Research Program includes a set of large community-based studies focused largely on cardiometabolic health carried out in the Old Order Amish (OOA) community of Lancaster, Pennsylvania.[2] The OOA population of Lancaster County, PA immigrated to the Colonies from Western Europe in the early 1700's. There are now over 30,000 OOA individuals in the Lancaster area, nearly all of whom can trace their ancestry back 12-14 generations to approximately 700 founders. Investigators at the University of Maryland School of Medicine have been studying the genetic determinants of cardiometabolic health in this population since 1993. To date, over 7,000 Amish adults have participated in one or more of our studies.

The Amish studies upon which these data are based were supported by NIH grants R01 AG18728, U01 HL072515, R01 HL088119, R01 HL121007, and P30 DK072488. WGS for “NHLBI

TOPMed: Genetics of Cardiometabolic Health in the Amish" (phs000956) was performed at the Broad Institute of MIT and Harvard (3R01HL121007-01S1).

Mt Sinai BioMe Biobank (BioMe, 9,857)

TOPMed dbGaP accession#: phs001644, Parent dbGaP accession#: phs000925

The Mount Sinai Institute for Personalized Medicine BioMe Biobank is a consented, EMR-linked medical care setting biorepository of the Mount Sinai Medical Center drawing from a population of over 70,000 inpatients and 800,000 outpatient visits annually.[3] The Mount Sinai Medical Center services diverse local communitiies of upper Manhattan, including Central Harlem (86% African American), East Harlem (88% Hispanic Latino), and Upper East Side (88% Caucasian/white) with broad health disparities. Biobank operations are fully integrated in clinical care processes, including direct recruitment from clinical sites waiting areas and phlebotomy stations by dedicated Biobank recruiters independent of clinical care providers, prior to or following a clinician standard of care visit. Recruitment currently occurs at a broad spectrum of over 30 clinical care sites.

The Mount Sinai BioMe Biobank has been supported by The Andrea and Charles Bronfman Philanthropies and in part by Federal funds from the NHLBI and NHGRI (U01HG00638001; U01HG007417; X01HL134588). WGS for "NHLBI TOPMed: Mount Sinai BioMe Biobank" (phs001644) was performed at the Baylor College of Medicine Human Genome Sequencing Center (HHSN268201600033I). We thank all participants in the Mount Sinai Biobank. We also thank all our recruiters who have assisted and continue to assist in data collection and management and are grateful for the computational resources and staff expertise provided by Scientific Computing at the Icahn School of Medicine at Mount Sinai.

Coronary Artery Risk Development in Young Adults (CARDIA, 3,054)

TOPMed dbGaP accession#: phs001612, Parent dbGaP accession#: phs000285

The Coronary Artery Risk Development in Young Adults Study (CARDIA) is a study examining the etiology and natural history of cardiovascular disease beginning in young adulthood.[4] In 1985-1986, a cohort of 5115 healthy black and white men and women aged 18-30 years were selected to have approximately the same number of people in subgroups of age (18-24 and 25-30), sex, race, and education (high school or less and more than high school) within each of four US Field Centers. These same participants were asked to participate in follow-up examinations during 1987-1988 (Year 2), 1990-1991 (Year 5), 1992-1993 (Year 7), 1995-1996 (Year 10), 2000-2001 (Year 15), 2005-2006 (Year 20), 2010-2011 (Year 25); and 2015-2016 (Year 30). A majority of the group has been examined at each of the follow-up examinations (91%, 86%, 81%, 79%, 74%, 72%, 72%, and 71%, respectively). In addition to the follow-up examinations, participants are contacted regularly for the ascertainment of information on out-patient procedures and hospitalizations experienced between contacts.

The Coronary Artery Risk Development in Young Adults Study (CARDIA) is conducted and supported by the National Heart, Lung, and Blood Institute (NHLBI) in collaboration with the University of Alabama at Birmingham (HHSN268201800005I & HHSN268201800007I), Northwestern University (HHSN268201800003I), University of Minnesota (HHSN268201800006I), and Kaiser Foundation Research Institute (HHSN268201800004I). CARDIA was also partially supported by the Intramural Research Program of the National Institute on Aging (NIA) and an intra-agency agreement between NIA and NHLBI (AG0005). WGS for "NHLBI TOPMed: Coronary Artery Risk Development in Young Adults" (phs001612) was performed at the Baylor College of Medicine Human Genome Sequencing Center (HHSN268201600033I).

Cleveland Family Study (CFS, 577)

TOPMed dbGaP accession#: phs000954, Parent dbGaP accession#: phs000284

The Cleveland Family Study (CFS) is the largest family-based study of sleep apnea, comprising of 2,284 individuals (46% African American) from 361 families studied up to 4 occasions

over 16 years, 1990-2006.[5, 6, 7, 8] Index probands (n=275) were recruited from 3 area hospital sleep labs if they had a confirmed diagnosis of sleep apnea and at least 2 first-degree relatives available to be studied. In the first 5 years of the study, neighborhood control probands (n=87) with at least 2 living relatives available for study were selected at random from a list provided by the index family and also studied. All available first-degree relatives and spouses of the case and control probands also were recruited. Second-degree relatives, including half-sibs, aunts, uncles and grandparents, were also included if they lived near the first-degree relatives (cases or controls), or if the family had been found to have two or more relatives with sleep apnea. Blood was sampled and DNA isolated for participants seen in the last two exam cycles (n=1,447).

CFS is supported by grants from the NHLBI (HL046389, HL113338, and 1R35HL135818). WGS for “NHLBI TOPMed: Cleveland Family Study - WGS Collaboration” (phs000954) was performed at the University of Washington Northwest Genomics Center (3R01HL098433-05S1 and HHSN268201600032I).

<u>Cardiovascular Health Study (CHS, 2,773)</u>

TOPMed dbGaP accession#: phs001368, Parent dbGaP accession#: phs000287

The Cardiovascular Health Study (CHS) originated in 1988 and is a study of risk factors for development and progression of coronary heart disease and stroke in people aged 65 years and older.[9, 10, 11] The 5,888 study participants were recruited from four U.S. communities and have undergone extensive clinic examinations for evaluation of markers of subclinical cardiovascular disease. The original cohort totaled 5,201 participants. A new cohort was recruited in 1992. The 687 participants in the new cohort are predominately African-American and were recruited at three of the four field centers. Starting in 1989, and continuing through 1999, participants underwent annual extensive clinical examinations. Measurements included traditional risk factors such as blood pressure and lipids as well as measures of subclinical disease, including echocardiography of the heart, carotid ultrasound, and cranial magnetic-resonance imaging (MRI). At six-month intervals between clinic visits, and once clinic visits ended, participants were contacted by phone to ascertain hospitalizations and health status. The main outcomes are coronary heart disease (CHD), angina, heart failure (HF), stroke, transient ischemic attack (TIA), claudication, and mortality. Participants continue to be contacted by phone every 6 months.

This CHS research was supported by NHLBI contracts HHSN268201200036C, HHSN268200800007C, HHSN268201800001C, N01HC55222, N01HC85079, N01HC85080, N01HC85081, N01HC85082, N01HC85083, N01HC85086; and NHLBI grants U01HL080295, R01HL087652, R01HL105756, R01HL103612, R01HL120393, R01HL130114, and R01 HL059367, with additional contribution from the National Institute of Neurological Disorders and Stroke (NINDS). Additional support was provided through R01AG023629 from the National Institute on Aging (NIA). A full list of principal CHS investigators and institutions can be found at CHS-NHLBI.org. WGS for “NHLBI TOPMed: Cardiovascular Health Study” (phs001368) was performed at the Baylor College of Medicine Human Genome Sequencing Center (3U54HG003273-12S2, HHSN268201500015C, and HHSN268201600033I). The content is solely the responsibility of the authors and does not necessarily represent the official views of the National Institutes of Health.

<u>Diabetes Heart Study (DHS, 365)</u>

TOPMed dbGaP accession#: phs001412, Parent dbGaP accession#: phs001012

The Diabetes Heart Study (DHS) is a family-based study enriched for type 2 diabetes (T2D).[12] The cohort included 1443 European American and African American participants from 564 families with multiple cases of type 2 diabetes. The cohort was recruited between 1998 and 2006. Participants were extensively phenotyped for measures of subclinical CVD and other known CVD risk factors. Primary outcomes were quantified burden of vascular calcified plaque in the coronary artery, carotid artery, and abdominal aorta all determined from non-contrast computed tomography scans.

This work was supported by R01 HL92301, R01 HL67348, R01 NS058700, R01 AR48797, R01 DK071891, R01 AG058921, the General Clinical Research Center of the Wake Forest University School of Medicine (M01 RR07122, F32 HL085989), the American Diabetes Association, and a pilot grant from the Claude Pepper Older Americans Independence Center of Wake Forest University Health Sciences (P60 AG10484). WGS for "NHLBI TOPMed: Diabetes Heart Study" (phs001412) was performed at the Broad Institute of MIT and Harvard (HHSN268201500014C).

Framingham Heart Study (FHS, 3,990)

TOPMed dbGaP accession#: phs000974, Parent dbGaP accession#: phs000007

The Framingham Heart Study (FHS) is a prospective cohort study of 3 generations of subjects who have been followed up to 65 years to evaluate risk factors for cardiovascular disease.[13, 14, 15, 16] Its large sample of ~15,000 men and women who have been extensively phenotyped with repeated examinations make it ideal for the study of genetic associations with cardiovascular disease risk factors and outcomes. DNA samples have been collected and immortalized since the mid-1990s and are available on ~8000 study participants in 1037 families. These samples have been used for collection of GWAS array data and exome chip data in nearly all with DNA samples, and for targeted sequencing, deep exome sequencing and light coverage whole genome sequencing in limited numbers. Additionally, mRNA and miRNA expression data, DNA methylation data, metabolomics and other 'omics data are available on a sizable portion of study participants. This project will focus on deep whole genome sequencing (mean 30X coverage) in ~4100 subjects and imputed to all with GWAS array data to more fully understand the genetic contributions to cardiovascular, lung, blood and sleep disorders.

FHS acknowledges the support of contracts NO1-HC-25195 and HHSN268201500001I from the National Heart, Lung and Blood Institute and grant supplement R01 HL092577-06S1 for this research. WGS for "NHLBI TOPMed: Whole Genome Sequencing and Related Phenotypes in the Framingham Heart Study" (phs000974) was performed at the Broad Institute of MIT and Harvard (HHSN268201500014C, 3R01HL092577-06S1, and 3U54HG003067-12S2). We also acknowledge the dedication of the FHS study participants without whom this research would not be possible.

Genetic Epidemiology Network of Arteriopathy (GENOA, 1,044)

TOPMed dbGaP accession#: phs001345, Parent dbGaP accession#: phs001238

The Genetic Epidemiology Network of Arteriopathy (GENOA) is one of four networks in the NHLBI Family-Blood Pressure Program (FBPP).[17] GENOA's long-term objective is to elucidate the genetics of target organ complications of hypertension, including both atherosclerotic and arteriolosclerotic complications involving the heart, brain, kidneys, and peripheral arteries.[18] The longitudinal GENOA Study recruited European-American and African-American sibships with at least 2 individuals with clinically diagnosed essential hypertension before age 60 years. All other members of the sibship were invited to participate regardless of their hypertension status. Participants were diagnosed with hypertension if they had either 1) a previous clinical diagnosis of hypertension by a physician with current anti-hypertensive treatment, or 2) an average systolic blood pressure ≥ 140 mm Hg or diastolic blood pressure ≥ 90 mm Hg based on the second and third readings at the time of their clinic visit. Only participants of the African-American Cohort were sequenced through TOPMed.

Support for GENOA was provided by the National Heart, Lung and Blood Institute (HL054457, HL054464, HL054481, and HL087660) of the National Institutes of Health. WGS for "NHLBI TOPMed: Genetic Epidemiology Network of Arteriopathy" (phs001345) was performed at the Broad Institute of MIT and Harvard (HHSN268201500014C) and the University of Washington Northwest Genomics Center (3R01HL055673-18S1).

Genetics of Lipid-Lowering Drugs and Diet Network (GOLDN, 924)

TOPMed dbGaP accession#: phs001359, Parent dbGaP accession#: phs000741

The Genetics of Lipid-Lowering Drugs and Diet Network (GOLDN) study was initiated to assess how genetic factors interact with environmental (diet and drug) interventions to influence blood levels of triglycerides and other atherogenic lipid species and inflammation markers (registered at clinicaltrials.gov, number NCT00083369).[19] The study recruited Caucasian participants primarily from three-generational pedigrees from two NHLBI Family Heart Study (FHS) field centers (Minneapolis, MN and Salt Lake City, UT).[20] Only families with at least two siblings were recruited and only participants who did not take lipid-lowering agents (pharmaceuticals or nutraceuticals) for at least 4 weeks prior to the initial visit were included. The diet intervention followed the protocol of Patsch et al.[21] The whipping cream (83% fat) meal had 700 Calories/m2 body surface area (2.93 mJ/m2 body surface area): 3% of calories were derived from protein (instant nonfat dry milk) and 14% from carbohydrate (sugar). The ratio of polyunsaturated to saturated fat was 0.06 and the cholesterol content of the average meal was 240 mg. The mixture was blended with ice and flavorings. Blood samples were drawn immediately before (fasting) and at 3.5 and 6 hours after consuming the high-fat meal. The diet intervention was administered at baseline as well as after a 3-week treatment with 160 mg micronized fenofibrate. Participants were given the option to complete one or both (diet and drug) interventions. Of all participants, 1079 had phenotypic data and provided appropriate consent, and underwent whole genome sequencing through the TOPMed program.

GOLDN biospecimens, baseline phenotype data, and intervention phenotype data were collected with funding from National Heart, Lung and Blood Institute (NHLBI) grant U01 HL072524. WGS for “NHLBI TOPMed: Genetics of Lipid Lowering Drugs and Diet Network” (phs001359) was performed at the University of Washington Northwest Genomics Center (3R01HL104135-04S1 and R01 HL104135).

Genetic Epidemiology Network of Salt Sensitivity (GenSalt, 1,770)

TOPMed dbGaP accession#: phs001217, Parent dbGaP accession#: phs000784

The Genetic Epidemiology Network of Salt-Sensitivity (GenSalt) study, using a family feeding-study design, aims to identify genes which interact with dietary sodium and potassium intake to influence blood pressure in Han Chinese participants from rural north China.[22] The dietary intervention included a 7-day low-sodium feeding (51.3 mmol/day), a 7-day high-sodium feeding (307.8 mmol/day) and a 7-day high-sodium feeding with an oral potassium supplementation (60 mmol/day). Microsatellite markers for genome-wide linkage scan and single nucleotide polymorphism (SNP) markers in candidate genes will be genotyped. Overall, 3153 participants from 658 families were recruited for GenSalt. Whole genome sequencing has been conducted for 1,860 participants as a part of TOPMed.

GenSalt was supported by research grants (U01HL072507, R01HL087263, and R01HL090682) from the National Heart, Lung and Blood Institute, National Institutes of Health, Bethesda, MD. WGS for “NHLBI TOPMed: Genetic Epidemiology Network of Salt Sensitivity” (phs001217) was performed at the Baylor College of Medicine Human Genome Sequencing Center (HHSN268201500015C).

Genetic Studies of Atherosclerosis Risk (GeneSTAR, 1,755)

TOPMed dbGaP accession#: phs001218, Parent dbGaP accession#: phs000375

GeneSTAR began in 1982 as the Johns Hopkins Sibling and Family Heart Study, a prospective longitudinal family-based study conducted originally in healthy adult siblings of people with documented early onset coronary disease under 60 years of age.[23, 24] Commencing in 2003, the siblings, their offspring, and the coparent of the offspring participated in a 2 week trial of aspirin 81 mg/day with pre and post ex vivo platelet function assessed using multiple agonists in whole blood and platelet rich plasma. Extensive additional cardiovascular testing and risk assessment was done at baseline and serially. Follow-up was carried out to determine incident cardiovascular disease, stroke, peripheral arterial disease, diabetes, cancer, and related comorbidities, from 5 to 30 years after study entry. The goal of several additional phenotyping and interventional substudies has been

to discover and amplify understanding of the mechanisms of atherogenic vascular diseases and attendant comorbidities.

GeneSTAR was supported by grants from the National Institutes of Health/National Heart, Lung, and Blood Institute (U01 HL72518, HL087698, HL49762, HL58625, HL071025, HL112064), the National Institutes of Health/National Institute of Nursing Research (NR0224103), and by a grant from the National Institutes of Health/National Center for Research Resources (M01-RR000052) to the Johns Hopkins General Clinical Research Center. WGS for "NHLBI TOPMed: Genetic Studies of Atherosclerosis Risk" (phs001218) was performed at the Broad Institute of MIT and Harvard (HHSN268201500014C), the Macrogen Corp. (3R01HL112064-04S1), and Illumina (R01HL112064).

Hispanic Community Health Study - Study of Latinos (HCHS/SOL, 7,391)

TOPMed dbGaP accession#: phs001395, Parent dbGaP accession#: phs000810

The Hispanic Community Health Study / Study of Latinos (HCHS/SOL) is a multi-center epidemiologic study in Hispanic/Latino populations to determine the role of acculturation in the prevalence and development of disease, and to identify risk factors playing a protective or harmful role in Hispanics/Latinos.[25] The goals of the HCHS/SOL include studying the prevalence and development of disease in Hispanics/Latinos, including the role of acculturation, and identifying disease risk factors that play protective or harmful roles in Hispanics/Latinos. A total of 16,415 persons of Cuban, Dominican, Mexican, Puerto Rican, Central American, and South American backgrounds were recruited through four Field Centers affiliated with San Diego State University, Northwestern University in Chicago, Albert Einstein College of Medicine in the Bronx area of New York, and the University of Miami. Seven additional academic centers serve as scientific and logistical support centers. Study participants aged 18-74 years took part in an extensive clinic exam and assessments to ascertain socio-demographic, cultural, environmental and biomedical characteristics. Annual follow-up interviews are conducted to determine a range of health outcomes.

The Hispanic Community Health Study/Study of Latinos was carried out as a collaborative study supported by contracts from the National Heart, Lung, and Blood Institute (NHLBI) to the University of North Carolina (N01-HC65233), University of Miami (N01-HC65234), Albert Einstein College of Medicine (N01-HC65235), Northwestern University (N01-HC65236), and San Diego State University (N01-HC65237). The following Institutes/Centers/Offices contribute to the HCHS/SOL through a transfer of funds to the NHLBI: National Center on Minority Health and Health Disparities, the National Institute of Deafness and Other Communications Disorders, the National Institute of Dental and Craniofacial Research, the National Institute of Diabetes and Digestive and Kidney Diseases, the National Institute of Neurological Disorders and Stroke, and the Office of Dietary Supplements. WGS for "NHLBI TOPMed: Hispanic Community Health Study - Study of Latinos" (phs001395) was performed at the Baylor College of Medicine Human Genome Sequencing Center (HHSN268201600033I).

Hypertension Genetic Epidemiology Network and Genetic Epidemiology Network of Arteriopathy (HyperGEN, 1,853)

TOPMed dbGaP accession#: phs001293, Parent dbGaP accession#: phs001293

The Hypertension Genetic Epidemiology Network Study (HyperGEN) - Genetics of Left Ventricular (LV) Hypertrophy is a familial study aimed to understand genetic risk factors for LV hypertrophy by conducting genetic studies of continuous traits from echocardiography exams.[26] The originating HyperGEN study aimed to understand genetic risk factors for hypertension.[27] HyperGEN recruited 470 multiply-affected population-based hypertensive AA sibships (N=1224 siblings) from 1996-1999. HyperGEN probands were ascertained by early onset hypertension (i.e., before 60 years); to participate, they had to have at least one hypertensive sibling who was also willing to participate. Data from detailed clinical exams as well as genotyping data for linkage studies, candidate gene studies and GWAS have been collected and is shared between HyperGEN and the ancillary HyperGEN - Genetics of LV Hypertrophy study.

The HyperGEN Study is part of the National Heart, Lung, and Blood Institute (NHLBI) Family Blood Pressure Program; collection of the data represented here was supported by grants U01 HL054472 (MN Lab), U01 HL054473 (DCC), U01 HL054495 (AL FC), and U01 HL054509 (NC FC). The HyperGEN: Genetics of Left Ventricular Hypertrophy Study was supported by NHLBI grant R01 HL055673 with whole-genome sequencing made possible by supplement -18S1. WGS for “NHLBI TOPMed: Hypertension Genetic Epidemiology Network” (phs001293) was performed at the University of Washington Northwest Genomics Center (3R01HL055673-18S1).

<u>Jackson Heart Study (JHS, 2,846)</u>
TOPMed dbGaP accession#: phs000964, Parent dbGaP accession#: phs000286

The purpose of the Jackson Heart Study (JHS) is to explore the reasons for heightened cardiovascular disease prevalence among African Americans and to uncover new approaches to reduce it. The JHS is a large, community-based, observational study whose 5,306 participants were recruited from among the non-institutionalized African-American adults from urban and rural areas of the three counties (Hinds, Madison, and Rankin) that make up the Jackson, MS, metropolitan statistical area (MSA).[28, 29, 30] The JHS design included participants from the Jackson ARIC study who had originally been recruited through random selection from a drivers' license registry. New JHS participants were chosen randomly from the Accudata America commercial listing, which provides householder name, address, zip code, phone number (if available), age group in decades, and family components. In addition, a family component was included in the JHS. The sampling frame for the family study was a participant in any one of the ARIC, random, or volunteer samples whose family size met eligibility requirements. Recruitment was limited to persons 35-84 years old except in the family cohort, where those 21 years old and above were eligible.

The Jackson Heart Study (JHS) is supported and conducted in collaboration with Jackson State University (HHSN268201800013I), Tougaloo College (HHSN268201800014I), the Mississippi State Department of Health (HHSN268201800015I) and the University of Mississippi Medical Center (HHSN268201800010I, HHSN268201800011I and HHSN268201800012I) contracts from the National Heart, Lung, and Blood Institute (NHLBI) and the National Institute on Minority Health and Health Disparities (NIMHD). WGS for “NHLBI TOPMed: The Jackson Heart Study” (phs000964) was performed at the University of Washington Northwest Genomics Center (HHSN268201100037C). The authors also wish to thank the staffs and participants of the JHS. The authors also wish to thank the staffs and participants of the JHS.

<u>Multi-Ethnic Study of Atherosclerosis (MESA, 5,283)</u>
TOPMed dbGaP accession#: phs001416, Parent dbGaP accession#: phs000209

The Multi-Ethnic Study of Atherosclerosis (MESA) is a study of the characteristics of subclinical cardiovascular disease (disease detected non-invasively before it has produced clinical signs and symptoms) and the risk factors that predict progression to clinically overt cardiovascular disease or progression of the subclinical disease.[31] MESA researchers study a diverse, population-based sample of 6,814 asymptomatic men and women aged 45-84. Thirty-eight percent of the recruited participants are white, 28 percent African-American, 22 percent Hispanic, and 12 percent Asian, predominantly of Chinese descent. Participants were recruited from six field centers across the United States: Wake Forest University, Columbia University, Johns Hopkins University, University of Minnesota, Northwestern University and University of California - Los Angeles. Each participant received an extensive exam and determination of coronary calcification, ventricular mass and function, flow-mediated endothelial vasodilation, carotid intimal-medial wall thickness and presence of echogenic lucencies in the carotid artery, lower extremity vascular insufficiency, arterial wave forms, electrocardiographic (ECG) measures, standard coronary risk factors, sociodemographic factors, lifestyle factors, and psychosocial factors. Selected repetition of subclinical disease measures and risk factors at follow-up visits allows study of the progression of disease. Blood samples have been assayed for putative biochemical risk factors and stored for case-control studies. DNA has been

extracted and lymphocytes cryopreserved (for possible immortalization) for study of candidate genes and possibly, genome-wide scanning, expression, and other genetic techniques. Participants are being followed for identification and characterization of cardiovascular disease events, including acute myocardial infarction and other forms of coronary heart disease (CHD), stroke, and congestive heart failure; for cardiovascular disease interventions; and for mortality.

Whole genome sequencing (WGS) for the Trans-Omics in Precision Medicine (TOPMed) program was supported by the National Heart, Lung and Blood Institute (NHLBI). WGS for “NHLBI TOPMed: Multi-Ethnic Study of Atherosclerosis (MESA)” (phs001416.v1.p1) was performed at the Broad Institute of MIT and Harvard (3U54HG003067-13S1). Centralized read mapping and genotype calling, along with variant quality metrics and filtering were provided by the TOPMed Informatics Research Center (3R01HL-117626-02S1). Phenotype harmonization, data management, sample-identity QC, and general study coordination, were provided by the TOPMed Data Coordinating Center (3R01HL-120393-02S1). MESA and the MESA SHARe project are conducted and supported by the National Heart, Lung, and Blood Institute (NHLBI) in collaboration with MESA investigators. Support for MESA is provided by contracts HHSN268201500003I, N01-HC-95159, N01-HC-95160, N01-HC-95161, N01-HC-95162, N01-HC-95163, N01-HC-95164, N01-HC-95165, N01-HC-95166, N01-HC-95167, N01-HC-95168, N01-HC-95169, UL1-TR-000040, UL1-TR-001079, UL1-TR-001420. MESA Family is conducted and supported by the National Heart, Lung, and Blood Institute (NHLBI) in collaboration with MESA investigators. Support is provided by grants and contracts R01HL071051, R01HL071205, R01HL071250, R01HL071251, R01HL071258, R01HL071259, and by the National Center for Research Resources, Grant UL1RR033176. The provision of genotyping data was supported in part by the National Center for Advancing Translational Sciences, CTSI grant UL1TR001881, and the National Institute of Diabetes and Digestive and Kidney Disease Diabetes Research Center (DRC) grant DK063491 to the Southern California Diabetes Endocrinology Research Center.

Massachusetts General Hospital Atrial Fibrillation Study (MGH_AF, 683)

TOPMed dbGaP accession#: phs001062, Parent dbGaP accession#: phs001001

The Massachusetts General Hospital (MGH) Atrial Fibrillation Study was initiated in 2001.[32, 33] The study has enrolled serial probands, unaffected and affected family members with atrial fibrillation. At enrollment participants undergo a structured interview to systematically capture their past medical history, AF treatments, and family history. An electrocardiogram is performed; the results of an echocardiogram are obtained; and blood samples are obtained. For the TOPMed whole genome sequencing project only early-onset atrial fibrillation cases were sequenced. Early-onset atrial fibrillation was defined as an age of onset prior to 66 years of age.

The MGH AF Study was supported by grants to Dr. Ellinor from the Fondation Leducq (14CVD01), the National Institutes of Health to Dr. Ellinor (1RO1HL092577, R01HL128914, K24HL105780) and Dr. Lubitz (1R01HL139731) and by grants from the American Heart Association to Dr. Ellinor (18SFRN34110082) and to Dr. Lubitz (18SFRN34250007). WGS for “NHLBI TOPMed: Massachusetts General Hospital Atrial Fibrillation Study” (phs001062) was performed at the Broad Institute of MIT and Harvard (3R01HL092577-06S1, 3U54HG003067-12S2, 3U54HG003067-13S1, and 3UM1HG008895-01S2)

San Antonio Family Study (SAFS, 617)

TOPMed dbGaP accession#: phs001215, Parent dbGaP accession#: phs000462

The San Antonio Family Heart Study is a complex pedigree-based mixed longitudinal study designed to identify low frequency or rare variants influencing susceptibility to cardiovascular disease, using whole genome sequence (WGS) information from 3,000 individuals in large Mexican American pedigrees from San Antonio, Texas.[34] The major objectives of this study are to identify low frequency or rare variants in and around known common variant signals for CVD, as well as to find novel low frequency or rare variants influencing susceptibility to CVD. The study began in 1991, and included

1,431 individuals in 42 extended families at baseline. Probands were 40 to 60 year old low-income Mexican Americans selected at random without regard to presence or absence of disease, almost exclusively from Mexican American census tracts in San Antonio, Texas. All first, second, and third -degree relatives of the proband and of the proband's spouse, aged 16 years or above, were eligible to participate in the study. 1,200 WGS at 30X WGS were obtained through Illumina funded by a supplement as part of the NHLBI's TOPMed program.

Collection of the San Antonio Family Study data was supported in part by National Institutes of Health (NIH) grants R01 HL045522, MH078143, MH078111 and MH083824; and whole genome sequencing of SAFS subjects was supported by U01 DK085524 and R01 HL113323. We are very grateful to the participants of the San Antonio Family Study for their continued involvement in our research programs. WGS for “NHLBI TOPMed: Whole Genome Sequencing to Identify Causal Genetic Variants Influencing CVD Risk - San Antonio Family Studies” (phs001215) was performed at Illumina (3R01HL113323-03S1 and R01HL113322).

Samoan Adiposity Study (Samoan, 1,182)

TOPMed dbGaP accession#: phs000972, Parent dbGaP accession#: phs000914

The research goal of the Samoan Adiposity Study is to identify genetic variation that increases susceptibility to obesity and cardiometabolic phenotypes among adult Samoans using genome-wide association (GWAS) methods.[35, 36] DNA from peripheral blood and phenotypic information were collected from 3,119 adult Samoans, 23 to 70 years of age. The participants reside throughout the independent nation of Samoa, which is experiencing economic development and the nutrition transition. Genotyping was performed with the Affymetrix Genome-Wide Human SNP 6.0 Array using a panel of approximately 900,000 SNPs. Anthropometric, fasting blood biomarkers and detailed dietary, physical activity, health and socio-demographic variables were collected. Whole genome sequencing of a subset was motivated by the opportunity to create a Samoan-specific reference panel for imputation into the larger parent study.

Data collection was funded by NIH grant R01-HL093093. WGS for “NHLBI TOPMed: Samoan Adiposity Study” (phs000972) was performed at the University of Washington Northwest Genomics Center (HHSN268201100037C and HHSN268201500016C). We thank the Samoan participants of the study and local village authorities. We acknowledge the support of the Samoan Ministry of Health and the Samoa Bureau of Statistics for their support of this research.

Taiwan Study of Hypertension using Rare Variants (THRV, 1,979)

TOPMed dbGaP accession#: phs001387, Parent dbGaP accession#: phs001387

The THRV-TOPMed study consists of three cohorts: The SAPPHIRe Family cohort (N=1,271), TSGH (Tri-Service General Hospital, a hospital-based cohort, N=160), and TCVGH (Taichung Veterans General Hospital, another hospital-based cohort, N=922), all based in Taiwan.[37, 38] 1,271 subjects were previously recruited as part of the NHLBI-sponsored SAPPHIRe Network (which is part of the Family Blood Pressure Program, FBPP). The SAPPHIRe families were recruited to have two or more hypertensive sibs, some families also with one normotensive/hypotensive sib. The two Hospital-based cohorts (TSGH and TCVGH) both recruited unrelated subjects with different recruitment criteria (matched with SAPPHIRe subjects for age, sex, and BMI category).

The Rare Variants for Hypertension in Taiwan Chinese (THRV) is supported by the National Heart, Lung, and Blood Institute (NHLBI) grant (R01HL111249) and its participation in TOPMed is supported by an NHLBI supplement (R01HL111249-04S1). THRV is a collaborative study between Washington University in St. Louis, LA BioMed at Harbor UCLA, University of Texas in Houston, Taichung Veterans General Hospital, Taipei Veterans General Hospital, Tri-Service General Hospital, National Health Research Institutes, National Taiwan University, and Baylor University. THRV is based (substantially) on the parent SAPPHIRe study, along with additional population-based and hospital-based cohorts. SAPPHIRe was supported by NHLBI grants (U01HL54527, U01HL54498) and Taiwan funds, and the other cohorts were supported by Taiwan funds. WGS for “NHLBI TOPMed: Taiwan

Study of Hypertension using Rare Variants" (phs001387) was performed at the Baylor College of Medicine Human Genome Sequencing Center (3R01HL111249-04S1, HHSN26820150015C)

Women's Health Initiative (WHI, 8,305)

TOPMed dbGaP accession#: phs001237, Parent dbGaP accession#: phs000200

The Women's Health Initiative (WHI) is a long-term national health study that has focused on strategies for preventing heart disease, breast and colorectal cancer, and osteoporotic fractures in postmenopausal women (clinicaltrials.gov NCT00000611).[39, 40, 41] The original WHI study included 161,808 postmenopausal women enrolled between 1993 and 1998. The Fred Hutchinson Cancer Research Center in Seattle, WA serves as the WHI Clinical Coordinating Center for data collection, management, and analysis of the WHI. The WHI has two major parts: a partial factorial randomized Clinical Trial (CT) and an Observational Study (OS); both were conducted at 40 Clinical Centers nationwide. The CT enrolled 68,132 postmenopausal women between the ages of 50-79 into trials testing three prevention strategies. If eligible, women could choose to enroll in one, two, or all three of the trial components. The components are: hormone therapy trials, dietary modification trial, and calcium / vitamin D trial. The Observational Study (OS) examines the relationship between lifestyle, environmental, medical and molecular risk factors and specific measures of health or disease outcomes. This component involves tracking the medical history and health habits of 93,676 women not participating in the CT. Recruitment for the observational study was completed in 1998 and participants were followed annually for 8 to 12 years.

The WHI program is funded by the National Heart, Lung, and Blood Institute, National Institutes of Health, U.S. Department of Health and Human Services through contracts HHSN268201600018C, HHSN268201600001C, HHSN268201600002C, HHSN268201600003C, and HHSN268201600004C. WGS for "NHLBI TOPMed: Women's Health Initiative" (phs001237) was performed at the Broad Institute of MIT and Harvard (HHSN268201500014C)

Supplementary Note 2. HUNT (external to TOPMed)

The Trøndelag Health Study (The HUNT Study) is a collaboration between HUNT Research Centre (Faculty of Medicine, Norwegian University of Science and Technology NTNU), Nord-Trøndelag County Council, Central Norway Health Authority, and the Norwegian Institute of Public Health. The HUNT-MI study, which comprises the genetic investigations of the HUNT Study, is a collaboration between investigators from the HUNT study, University of Michigan School of Public Health and Michigan Medicine. The K.G. Jebsen Center for Genetic Epidemiology is financed by Stiftelsen Kristian Gerhard Jebsen; Faculty of Medicine and Health Sciences, NTNU, Norwegian University of Science and Technology (NTNU) and Central Norway Regional Health Authority. We thank all HUNT study participants. C.J.W is funded by R35-HL138139.

Supplementary Note 3. UK Biobank (external to TOPMed)

The UK Biobank analyses were conducted using the UK Biobank resource under application 7089.

Supplementary Note 4. FinnGen (external to TOPMed)

The FinnGen project is funded by two grants from Business Finland (HUS 4685/31/2016 and UH 4386/31/2016) and eleven industry partners (AbbVie Inc, AstraZeneca UK Ltd, Biogen MA Inc, Celgene Corporation, Celgene International II Sàrl, Genentech Inc, Merck Sharp & Dohme Corp, Pfizer Inc., GlaxoSmithKline, Sanofi, Maze Therapeutics Inc., Janssen Biotech Inc). Following biobanks are acknowledged for delivering biobank samples to FinnGen: Auria Biobank (www.auria.fi/biopankki), THL Biobank (www.thl.fi/biobank), Helsinki Biobank (www.helsinginbiopankki.fi), Biobank Borealis of Northern Finland (https://www.ppshp.fi/Tutkimus-ja-opetus/Biopankki/Pages/Biobank-Borealis-briefly-

in-English.aspx), Finnish Clinical Biobank Tampere (www.tays.fi/en-US/Research_and_development/Finnish_Clinical_Biobank_Tampere), Biobank of Eastern Finland (www.ita-suomenbiopankki.fi/en), Central Finland Biobank (www.ksshp.fi/fi-FI/Potilaalle/Biopankki), Finnish Red Cross Blood Service Biobank (www.veripalvelu.fi/verenluovutus/biopankkitoiminta) and Terveystalo Biobank (www.terveystalo.com/fi/Yritystietoa/Terveystalo-Biopankki/Biopankki/). All Finnish Biobanks are members of BBMRI.fi infrastructure (www.bbmri.fi).

Patients and control subjects in FinnGen provided informed consent for biobank research, based on the Finnish Biobank Act. Alternatively, separate research cohorts, collected prior the Finnish Biobank Act came into effect (in September 2013) and start of FinnGen (August 2017), were collected based on study-specific consents and later transferred to the Finnish biobanks after approval by Fimea, the National Supervisory Authority for Welfare and Health. Recruitment protocols followed the biobank protocols approved by Fimea. The Coordinating Ethics Committee of the Hospital District of Helsinki and Uusimaa (HUS) approved the FinnGen study protocol Nr HUS/990/2017.

The FinnGen project is approved by Finnish Institute for Health and Welfare (THL), approval number THL/2031/6.02.00/2017, amendments THL/1101/5.05.00/2017, THL/341/6.02.00/2018, THL/2222/6.02.00/2018, THL/283/6.02.00/2019), Digital and population data service agency VRK43431/2017-3, VRK/6909/2018-3, the Social Insurance Institution (KELA) KELA 58/522/2017, KELA 131/522/2018, KELA 70/522/2019 and Statistics Finland TK-53-1041-17.

The Biobank Access Decisions for FinnGen samples and data utilized in FinnGen Data Freeze 4 include: THL Biobank BB2017_55, BB2017_111, BB2018_19, BB_2018_34, BB_2018_67, BB2018_71, BB2019_7 Finnish Red Cross Blood Service Biobank 7.12.2017, Helsinki Biobank HUS/359/2017, Auria Biobank AB17-5154, Biobank Borealis of Northern Finland_2017_1013, Biobank of Eastern Finland 1186/2018, Finnish Clinical Biobank Tampere MH0004, Central Finland Biobank 1-2017, and Terveystalo Biobank STB 2018001.

Supplementary Table 1. Baseline characteristics of TOPMed participants in discovery genetic association analyses by cohort.

Cohort	Total	Age (y)	F	Cholesterol (mg/dl)			TG (mg/dl)	Statin Rx	Reported Ancestry					
				Total	HDL	LDL			EUR	AFR	HIS	ASN	SAM	AI_AN
ARIC	7,991	53.9 (5.8)	4,463 (55.9%)	213.9 (41.5)	51.7 (17.1)	136.6 (38.6)	109.0 [78.0-156.0]	226 (2.8%)	6,180 (77.3%)	1811 (22.7%)	0 (0%)	0 (0%)	0 (0%)	0 (0%)
Amish	1,083	50.4 (16.9)	535 (49.4%)	211.9 (47.0)	55.9 (15.6)	140.4 (43.3)	63.0 [46.0-96.0]	35 (3.2%)	1,083 (100%)	0 (0%)	0 (0%)	0 (0%)	0 (0%)	0 (0%)
BioMe	9,857	52.8 (13.2)	5,892 (59.8%)	182.5 (44.4)	53.8 (18.2)	103.0 (35.5)	109.0 [74.0-161.0]	2322 (23.6%)	2,675 (27.1%)	2,664 (27.0%)	4,307 (43.7%)	211 (2.1%)	0 (0%)	0 (0%)
CARDIA	3,054	25.1 (3.6)	1,732 (56.7%)	177.3 (32.9)	53.3 (12.9)	109.6 (30.7)	62.0 [45.0-83.8]	0 (0%)	1,684 (55.1%)	1,370 (44.9%)	0 (0%)	0 (0%)	0 (0%)	0 (0%)
CFS	577	46.7 (16.5)	324 (56.2%)	167.6 (36.8)	43.2 (12.5)	99.9 (32.4)	101.0 [75.0-144.0]	91 (15.8%)	255 (44.2%)	322 (55.8%)	0 (0%)	0 (0%)	0 (0%)	0 (0%)
CHS	2,773	72.6 (5.4)	1,591 (57.4%)	211.8 (39.1)	54.0 (15.7)	130.2 (35.6)	123.0 [94.0-166.0]	141 (5.1%)	2,612 (94.2%)	161 (5.8%)	0 (0%)	0 (0%)	0 (0%)	0 (0%)
DHS	365	59.6 (8.9)	208 (56.9%)	180.0 (42.0)	48.6 (13.8)	106.4 (34.4)	101.0 [76.0-148.0]	189 (51.2%)	0 (0%)	365 (100%)	0 (0%)	0 (0%)	0 (0%)	0 (0%)
FHS	3,990	40.3 (10.9)	2,152 (53.9%)	198.1 (39.0)	53.5 (16.2)	120.7 (35.0)	89.0 [61.0-143.0]	158 (4.0%)	3,990 (100%)	0 (0%)	0 (0%)	0 (0%)	0 (0%)	0 (0%)
GENOA	1,044	56.2 (10.9)	726 (69.5%)	203.8 (45.9)	55.2 (18.1)	120.7 (42.1)	125.0 [95.0-169.3]	52 (5.0%)	0 (0%)	1,044 (100%)	0 (0%)	0 (0%)	0 (0%)	0 (0%)
GOLDN	924	47.6 (16.2)	484 (52.4%)	188.9 (38.3)	46.9 (13.1)	121.1 (31.0)	109.0 [72.0-170.3]	0 (0%)	924 (100%)	0 (0%)	0 (0%)	0 (0%)	0 (0%)	0 (0%)
GenSalt	1,770	39.3 (8.9)	834 (47.1%)	171.6 (33.5)	51.1 (11.3)	95.9 (27.2)	100.1 [75.0-147.9]	14 (0.8%)	0 (0%)	0 (0%)	0 (0%)	1,770 (100%)	0 (0%)	0 (0%)
GeneSTAR	1,755	41.5 (11.4)	1,037 (59.1%)	204.3 (44.1)	52.4 (15.2)	127.3 (39.7)	103.0 [72.0-150.0]	129 (7.4%)	978 (55.7%)	777 (44.3%)	0 (0%)	0 (0%)	0 (0%)	0 (0%)
HCHS/SOL	7,391	46.5 (13.95)	4298 (58.2%)	199.1 (44.4)	49.3 (13.2)	123.0 (37.4)	113.0 [78.0-163.0]	957 (13.0%)	0 (0%)	0 (0%)	7,391 (100%)	0 (0%)	0 (0%)	0 (0%)
HyperGEN	1,853	46.9 (12.8)	1,170 (63.1%)	194.7 (40.9)	53.5 (15.4)	119.7 (37.4)	91.0 [66.0-128.0]	90 (4.9%)	0 (0%)	1,853 (100%)	0 (0%)	0 (0%)	0 (0%)	0 (0%)
JHS	2,846	54.4 (12.96)	1,782 (62.6%)	198.1 (40.3)	51.3 (14.5)	125.8 (36.8)	89.0 [64.0-128.0]	319 (11.2%)	0 (0%)	2,846 (100%)	0 (0%)	0 (0%)	0 (0%)	0 (0%)
MESA	5,283	60.6 (9.7)	2,785 (52.7%)	194.3 (35.5)	51.3 (14.7)	117.8 (31.6)	108.0 [76.0-157.0]	900 (17.0%)	1,834 (34.7%)	1,830 (34.6%)	1019 (19.3%)	600 (11.4%)	0 (0%)	0 (0%)
MGH_AF	683	55.4 (10.3)	149 (21.8%)	182.4 (40.7)	50.4 (16.6)	107.4 (33.7)	103.0 [73.0-152.0]	190 (27.8%)	683 (100%)	0 (0%)	0 (0%)	0 (0%)	0 (0%)	0 (0%)
SAFS	617	50.2 (15.2)	382 (61.9%)	186.2 (39.9)	50.0 (15.1)	109.5 (32.9)	114.9 [81.0-165.9]	95 (15.4%)	0 (0%)	0 (0%)	617 (100%)	0 (0%)	0 (0%)	0 (0%)
Samoan	1,182	44.7 (11.3)	716 (60.6%)	198.7 (36.3)	45.1 (10.7)	129.4 (32.4)	106.0 [77.0-148.0]	0 (0%)	0 (0%)	0 (0%)	0 (0%)	0 (0%)	1,182 (100%)	0 (0%)
THRV	1,979	51.5 (9.8)	1,012 (51.1%)	187.8 (39.3)	47.3 (14.3)	115.2 (35.8)	110.0 [76.3-159.0]	84 (4.3%)	0 (0%)	0 (0%)	0 (0%)	1,979 (100%)	0 (0%)	0 (0%)
WHI	8,305	66.8 (6.8)	8,305 (100%)	230.4 (40.6)	55.4 (15.0)	145.9 (37.6)	129.5 [93.0-181.0]	764 (9.2%)	6,615 (79.7%)	1,388 (16.7%)	98 (1.2%)	154 (1.8%)	0 (0%)	50 (0.6%)
**ALL**	65,322	52.4 (14.9)	40,577 (62.1%)	200.0 (44.0)	52.2 (15.7)	122.5 (38.6)	106.0 [73.9-155.7]	6756 (10.3%)	29,513 (45.2%)	16,431 (25.2%)	13,432 (20.6%)	4,714 (7.2%)	1,182 (1.8%)	50 (0.08%)

Age and cholesterol values are expressed as mean(SD), triglyceride values are expressed as median[Q1-Q3], and count data are expressed as N(%).
AFR = African; AI_AN = American Indian/Alaskan Native/Native American; ASN = Asian; EUR = European; F = female; HDL = high-density lipoprotein; HIS = Hispanic; LDL = low-density lipoprotein; Rx = prescription; SAM = Samoan; TG = triglycerides.

Supplementary Table 2. Baseline characteristics of TOPMed participants in discovery genetic association analyses by reported ancestry.

Ancestry	Total	Age	Sex	Cholesterol			TG	Lipid Rx
				Total	HDL	LDL		
EUR	29,513 (45.2%)	55.1 (15.5)	18,605 (63.0%)	206.8 (43.4)	53.2 (16.3)	127.9 (38.4)	109.0 [75.0-161.0]	2,562 (8.7%)
AFR	16,431 (25.2%)	51.3 (14.5)	10,799 (65.7%)	196.7 (44.1)	53.7 (16.0)	121.6 (39.9)	91.0 [65.0-131.0]	1,756 (10.7%)
HIS	13,432 (20.6%)	49.9 (14.0)	7,983 (59.4%)	193.9 (45.0)	49.3 (14.1)	116.6 (37.5)	119.0 [82.0-173.0]	2,193 (16.3%)
ASN	4,714 (7.2%)	48.4 (12.6)	2,424 (51.4%)	182.9 (37.6)	49.9 (13.7)	107.3 (32.9)	107.1 [77.0-157.0]	243 (5.2%)
SAM	1,182 (1.8%)	44.7 (11.3)	716 (60.6%)	198.7 (36.3)	45.1 (10.7)	129.4 (32.4)	106.0 [77.0-148.0]	0 (0%)
AI_AN	50 (0.1%)	64.1 (7.3)	50 (100%)	227.2 (46.9)	52.4 (12.0)	136.2 (37.5)	160.0 [116.0-215.8]	2 (4%)
**ALL**	65,322 (100%)	52.4 (14.9)	40,577 (62.1%)	199.8 (44.0)	52.2 (15.7)	122.5 (38.6)	106 [73.9-155.7]	6,756 (10.3%)

Age and cholesterol values are expressed as mean(SD), triglyceride values are expressed as median[Q1-Q3], and count data are expressed as N(%).
AFR = African; AI_AN = American Indian/Alaskan Native/Native American; ASN = Asian; EUR = European; HDL = high-density lipoprotein; HIS = Hispanic; LDL = low-density lipoprotein; Rx = prescription; SAM = Samoan; TG = triglycerides.

Supplementary Table 3. **Chromosome X variant characteristics per sample by TOPMed cohort and sex.**

	Females					Males			
	**N**	**Ti/Tv**	**Het/Hom**	**Total variants**	**Singletons**	**N**	**Ti/Tv**	**Total variants**	**Singletons**
ARIC	4463	2.031 (0.011)	1.662 (0.330)	128962 (22075)	196 (95)	3528	2.030 (0.012)	87747 (11517)	7 (4)
Amish	535	2.030 (0.008)	1.333 (0.203)	114175 (3687)	9 (18)	548	2.026 (0.011)	81842 (2175)	0 (1)
BioMe	5892	2.026 (0.011)	1.917 (0.403)	142149 (21503)	243 (231)	3965	2.029 (0.012)	90754 (11922)	8 (7)
CARDIA	1732	2.027 (0.011)	1.821 (0.384)	140249 (25222)	255 (103)	1322	2.027 (0.012)	93616 (14563)	9 (5)
CFS	324	2.026 (0.011)	1.908 (0.392)	145414 (25038)	111 (100)	253	2.027 (0.013)	96392 (14900)	5 (5)
CHS	1591	2.032 (0.010)	1.547 (0.227)	119913 (1247`)	209 (113)	1182	2.030 (0.012)	84238 (6290)	7 (4)
DHS	208	2.020 (0.009)	2.186 (0.214)	165764 (8195)	160 (125)	157	2.023 (0.011)	111255 (6869)	7 (5)
FHS	2152	2.034 (0.010)	1.507 (0.123)	116694 (2675)	124 (118)	1838	2.033 (0.013)	80488 (3384)	5 (4)
GENOA	726	2.021 (0.008)	2.140 (0.223)	167349 (5555)	63 (78)	318	2.023 (0.011)	109934 (6415)	5 (4)
GOLDN	484	2.034 (0.010)	1.493 (0.113)	116537 (2640)	95 (104)	440	2.030 (0.011	80938 (2742)	4 (4)
GenSalt	834	2.026 (0.008)	1.185 (0.083)	116311 (2106)	341 (266)	936	2.030 (0.012)	84905 (3522)	9 (7)
GeneSTAR	1037	2.027 (0.012)	1.828 (0.384)	140216 (25121)	110 (112)	718	2.028 (0.012)	91960 (14216)	5 (5)
HCHS/SOL	4298	2.028 (0.010)	1.837 (0.410)	132319 (14778)	232 (171)	3093	2.029 (0.012)	89769 (8495)	8 (6)
HyperGEN	1170	2.020 (0.008)	2.137 (0.210)	167477 (5639)	126 (109)	683	2.023 (0.011)	108792 (7225)	7 (5)
JHS	1782	2.020 (0.009)	2.151 (0.238)	166954 (6645)	142 (108)	1064	2.022 (0.016)	109415 (7307)	7 (5)
MESA	2785	2.027 (0.011)	1.769 (0.435)	137034 (23357)	259 (192)	2498	2.02735 (0.011)	92546 (13099)	9 (6)
MGH_AF	149	2.035 (0.008)	1.513 (0.154)	117076 (3691)	276 (133)	534	2.029 (0.012)	82383 (4554)	12 (35)
SAFS	382	2.028 (0.010)	1.492 (0.269)	121218 (6099)	110 (117)	235	2.031 (0.013)	85006 (4250)	5 (5)
Samoan	716	2.026 (0.009)	1.096 (0.171)	115864 (3715)	122 (94)	466	2.028 (0.0103)	85265 (2786)	5 (5)
THRV	1012	2.027 (0.009)	1.176 (0.138)	116066 (3606)	299 (221)	967	2.0271 (0.011)	86085 (3357)	15 (59)
WHI	8305	2.032 (0.011)	1.617 (0.315)	125013 (18460)	255 (120)	0	NA	NA	NA
**TOTAL**	**40577**	**2.028 (0.011)**	**1.722 (0.404)**	**133255 (22455)**	**212 (166)**	**24745**	**2.02835 (0.012)**	**90117 (12166)**	**8 (14)**

Each continuous metric is expressed as mean (standard deviation)
Het/Hom = heterozygous to homozygous genotype counts ratio; Ti/Tv = transition to transversion ratio

Supplementary Table 4. Chromosome X variant counts by minor allele frequency bins

By Study	Total Samples	MAC 1	MAC 2	MAC 3	MAC 4 – MAF 0.1%	MAF 0.1-1.0%	MAF 1.0-5.0%	MAF 5.0%-50%	Total variants
ARIC	7,991	2051998	1143815	394437	860633	577744	202822	229316	5460753
Amish	1,083	22138	6626	6680	315	59658	62967	188117	339771
BioMe	9,857	2680736	1321112	433579	1132870	631761	241641	209571	6706481
CARDIA	3,054	1389391	695923	211048	279006	587968	242094	266061	3671461
CFS	577	221372	158424	108510	141	583262	258958	226277	1345648
CHS	2,773	1115677	553667	181076	189200	504375	125243	197897	2867100
DHS	365	277382	212828	88139	0	750407	263009	329041	1342457
FHS	3,990	456418	418354	306156	390861	330507	93721	192568	2188568
GENOA	1,044	383318	259601	145846	339	612628	269346	331328	1854909
GOLDN	924	169868	128692	80658	816	446607	97517	191126	905820
GenSalt	1,770	329836	322992	166274	1331	422154	79343	171831	1504385
GeneSTAR	1,755	413416	305167	213395	217	713325	244469	210789	2155445
HCHS/SOL	7,391	1671675	966190	398811	950681	601789	200875	235186	5025198
HyperGEN	1,853	535092	377520	178153	326	695870	266958	330064	2383533
JHS	2,846	867925	487481	221111	247702	638633	265387	329783	3057921
MESA	5,283	1790587	1099057	356976	708748	634608	232612	201206	5077793
MGH_AF	683	211977	332494	46424	833	530222	97302	192815	1032912
SAFS	617	192778	115913	82326	553	422217	121482	193163	929868
Samoan	1,182	217671	94428	50323	2824	176247	58251	170138	719369
THRV	1,979	424305	325744	128603	1576	440456	80582	170204	1571286
WHI	8,305	4006130	846434	356194	826728	569312	179812	217275	7001877
**Across All Studies**	**65,322**	**7375647**	**3867043**	**1776661**	**5792496**	**620628**	**220071**	**245123**	**19897667**

Supplementary Table 5. Chromosome X variants showing at least suggestive evidence of association with blood lipids in TOPMed and the evidence of replication in UK Biobank and HUNT.

	TOPMed						UK Biobank Whites					HUNT			
Trait	rsID	GRCh38 Chr:Pos:Ref:Alt	AAF	Beta	SE	P	rsID	AAF	Beta	SE	P	AAF	Beta	SE	P
TC	**rs5942634**	**chrX:110464413:A:T**	**0.344**	**-1.95**	**0.237**	**2.00E-16**	**rs5942634**	**0.387**	**-1.194**	**0.0819**	**3.59E-48**	0.382	-1.2410	0.198	3.4E-10
	rs185814586	chrX:154690417:C:G	0.0391	-3.627	0.593	9.74E-10	rs185814586	0.003	9.803	6.9291	1.57E-01	N/A			
	rs145493495	chrX:93454733:C:T	0.0062	-7.697	1.406	4.40E-08	rs145493495	0.0004	-6.973	9.9419	0.4831	N/A			
	rs138747372	chrX:32141527:A:G	0.0046	-8.221	1.626	4.28E-07	rs138747372	0.0003	37.499	20.4953	0.0673	N/A			
	rs781790319	chrX:143911300:G:A	0.0006	21.53	4.294	5.33E-07	rs781790319	0.00006	17.729	8.5473	0.0381	N/A			
Log(TG)	**rs5985504**	**chrX:110605767:T:C**	**0.5673**	**0.0188**	**0.0029**	**4.16E-11**	**rs5985504**	**0.602**	**0.0116**	**0.00099**	**2.15E-31**	**0.601**	0.0123	0.002	4.30E-07
	rs183294530	chrX:94076540:G:A	0.0023	0.1494	0.0273	4.38E-08	rs183294530	0.002	-0.0059	0.01127	5.98E-01	N/A			
	rs921187255	chrX:54528114:C:T	0.000173	-0.536	0.1034	2.18E-07	no proxy					N/A			
	rs777673958	chrX:70371648:G:T	0.00098	0.2123	0.0411	2.40E-07	no proxy					N/A			
	rs187325394	chrX:134877208:A:G	0.0004	-0.3185	0.0629	4.02E-07	rs187325394	0.0009	-0.1816	0.0944	5.44E-02	N/A			
		chrX:117213036:C:T	0.0002	-0.5098	0.1016	5.22E-07	no proxy					N/A			
	rs754747891	chrX:79229651:T:A	0.0003	0.4158	0.0835	6.30E-07	rs749043821	0.00005	N/A			N/A			
	rs942975446	chrX:47314065:C:T	0.0002	0.4987	0.1016	9.21E-07	no proxy					N/A			
HDL-C	rs988403543	chrX:111037797:A:T	0.0002	14.466	2.57573	1.95E-08	no proxy					N/A			
	rs902277370	chrX:104738300:G:T	0.0002	13.89	2.64339	1.48E-07	no proxy					N/A			
	rs966434643	chrX:85687610:C:T	0.00015	14.97	2.96778	4.56E-07	no proxy					N/A			
	**rs5935802**	**chrX:14723826:C:T**	**0.442**	**0.4044**	**0.08221**	**8.69E-07**	**rs5935802**	**0.564**	**0.1228**	**0.0277**	**9.44E-06**	**0.57**	0.0685	0.062	0.27
LDL-C	**rs5942648**	**chrX:110545499:G:A**	**0.3827**	**-1.529**	**0.215**	**1.19E-12**	**rs5942648**	**0.391**	**-1.016**	**0.0643**	**3.81E-56**	**0.384**	-0.8934	0.181	7.40E-07
	rs201851580	chrX:154959385:C:CA	0.0354	-3.548	0.574	6.42E-10	rs201851580	0.004	0.472	1.261	7.08E-01	N/A			
	rs750131104	chrX:65851068:T:C	0.0041	8.103	1.559	2.01E-07	rs750131104	0.0002	-3.676	32.209	9.09E-01	N/A			
	rs182564432	chrX:145894241:T:C	0.0007	-19.815	3.843	2.52E-07	rs182564432	0.00003	N/A			N/A			

Top chromosome X variants at loci showing at least suggestive evidence (*P*<1x10^{-6}) of association with lipids in TOPMed are displayed. Distinct loci are defined by +/- 400 kb window surrounding the lowest *P*. If variants prioritized by TOPMed chromosome X sequencing are not genotyped or imputed in UK Biobank chromosome X array genotyping, suitable proxies are used when present (LD r^2 > 0.8). Effect estimates for triglycerides are on the natural log scale.

AAF = alternative allele frequency; LD = linkage disequilibrium; *P* = p-value; SE = standard error; TOPMed = Trans-Omics for Precision Medicine

Supplementary Table 6. **Baseline characteristics of UK Biobank participants in replication analyses.**

Metric	Value
Age (y)	56.9 (7.9)
Sex	179,963 (46.1%)
European ancestry	376,358 (96.4%)
Cholesterol (mg/dl)	
Total	221.1 (44.3)
HDL	56.1 (14.8)
LDL	138.1 (33.7)
Triglycerides (mg/dl)	132.6 [93.6-191.5]
Statin Rx	64,004 (16.4%)
BMI (kg/m^2)	27.4 (4.8)
Systolic blood pressure (mmHg)	140.2 (19.7)
Diastolic blood pressure (mmHg)	82.3 (10.7)
Current smoker	39,736 (10.2%)
Diabetes mellitus type 2	25,349 (6.5%)
Coronary artery disease	18,204 (4.8%)

Continuous values are presented as mean (standard deviation) except for triglycerides which is given as median (Q1-Q3) due to the skewness of the triglyceride distribution. Categorical data are presented as count (percentage).
BMI = body mass index; HDL = high-density lipoprotein; LDL = low-density lipoprotein

Supplementary Table 7. Baseline characteristics of HUNT participants in replication analyses.

Metric	Value
Age (y)	47.9 (16.9)
Sex	36,907 (53.0%)
European ancestry	69,635 (100%)
Cholesterol (mg/dl)	
Total	222.5 (48.6)
HDL	52.8 (14.7)
LDL	139.9 (42.8)
Triglycerides (mg/dl)	152.6 (99.9)
Statin Rx	NA
BMI (kg/m^2)	26.3 (4.2)
Systolic blood pressure (mmHg)	135.1 (21.1)
Diastolic blood pressure (mmHg)	78.5 (12.5)
Current smoker	6,480
Diabetes mellitus type 2	5,382 (7.7%)
Coronary artery disease	7,710 (11.6%)

Continuous values are presented as mean (standard deviation) and categorical data are presented as count (percentage).
BMI = body mass index; HDL = high-density lipoprotein; LDL = low-density lipoprotein

Supplementary Table 8. **Pairwise linkage disequilibrium of chrXq23 variants associated with lipid levels in TOPMed.**

	rs5985504	rs5942634	rs5942648
**rs5985504**	1.00	0.61	0.73
**rs5942634**	0.61	1.00	0.84
**rs5942648**	0.73	0.84	1.00

Supplementary Table 9. **Lipid effect estimates within TOPMed by sex for chrXq23 associated variants**

		Males		Females		I^2, P for heterogeneity
**Variant**	**Trait**	**Beta±SE**	**P**	**Beta±SE**	**P**	
**rs5985504**	Triglycerides	-0.025±0.0041	$7.3\text{x}10^{-10}$	-0.01±0.0040	$6.3\ \text{x}10^{-3}$	I^2 = 85.4%, P = 0.0088
**rs5942634**	Total cholesterol	-1.91±0.31	$1.8\text{x}10^{-9}$	-1.84±0.34	$6.7\text{x}10^{-8}$	I^2 = 0%, P = 0.8791
**rs5942648**	LDL-C	-1.53±0.29	$1.0\text{x}10^{-7}$	-1.74±0.32	$3.4\text{x}10^{-6}$	I^2 = 0%, P = 0.6268

Effect estimate for Total cholesterol and LDL-C are in mg/dl units while effect estimates for triglycerides are in log(mg/dl) units. Linear regression association analyses were adjusted for age, age^2, batch, and principal components, as well as study-specific covariates where appropriate. P-values presented are two-sided without further adjustment for this secondary analyses.
LDL-C = low-density lipoprotein cholesterol; SE = standard error.

Supplementary Table 10. **Ancestry-specific allele frequencies and effect estimates for associated chrXq23 variants with total cholesterol in TOPMed.**

Ancestry	AN	rs5942634-T				rs5942648-A				rs5985504-T			
		AF	Beta	SE	P	AF	Beta	SE	P	AF	Beta	SE	P
ASN	402	0.02	-2.46	2.22	0.27	0.02	-2.46	2.22	0.27	0.02	-2.03	2.31	0.38
AFR	25192	0.38	-1.44	0.46	1.7×10^{-3}	0.50	-1.52	0.44	5.98×10^{-4}	0.67	-1.74	0.47	2.50×10^{-4}
HIS	16382	0.31	-1.99	0.57	4.6×10^{-4}	0.34	-1.84	0.55	8.64×10^{-3}	0.37	-1.75	0.55	1.52×10^{-3}
SAM	64	0.01	-3.01	5.83	0.61	0.01	-3.01	5.83	0.61	0.01	-1.37	5.78	0.81
EUR	47694	0.41	-2.04	0.32	1.06×10^{-10}	0.41	-1.95	0.31	5.79×10^{-10}	0.41	-1.85	0.31	4.40×10^{-9}
Test of Heterogeneity across ancestries		I^2 = 0.0%, P = 0.860				I^2 = 0.0%, P = 0.943				I^2 = 0.0%, P = 0.999			

Linear regression association analyses were adjusted for age, age^2, sex, batch, and principal components, as well as study-specific covariates where appropriate. P-values presented are two-sided without further adjustment for this secondary analyses. AF = allele frequency; AFR = African; AN = allele number; ASN = East Asian; EUR = European; HIS = Hispanic; SAM = Samoan; SE = standard error.

Supplementary Table 11. **Results for association with total cholesterol in conditional analysis with TOPMed samples.**

SNP	Ref	Alt	Beta	SE	P
**SNPs individually in model**					
rs5942634	A	T	-1.950	0.237	2.00E-16
rs5985471	C	T	-1.714	0.235	3.21E-13
rs5942937	T	G	-1.623	0.239	1.02E-11
rs5943057	T	G	-1.685	0.246	7.11E-12
**Adjusting for rs5942634**					
rs5985471	C	T	-0.176	0.387	0.649
rs5942937	T	G	0.291	0.426	0.4952
rs5943057	T	G	-0.309	0.363	0.3937
**Adjusting for rs5985471, rs5942937, and rs5943057**					
rs5942634	A	T	-1.614	0.453	0.000368

Linear regression association analyses were adjusted for age, age^2, sex, batch, and principal components, as well as study-specific covariates where appropriate. P-values presented are two-sided without further adjustment for this secondary analyses.

Supplementary Table 12. **Baseline characteristics of FinnGen participants for coronary heart disease analyses.**

Metric	Value
Age (y)	59.39 (17.35)
Sex	76,538 (43.27%)
European ancestry	176,899 (100%)
Hypercholesterolemia	10,041 (5.88%)
Cholesterol (mg/dl)	
Total	NA
HDL	NA
LDL	NA
Triglycerides (mg/dl)	NA
Statin Rx	53,518 (30.25%)
BMI (kg/m^2)	27.16 (5.26)
Hypertension	43,545 (24.62%)
Current smoker	948 (0.82%)
Diabetes mellitus type 2	23,364 (13.65)
Coronary artery disease	16,631 (9.4%)

Continuous values are presented as mean (standard deviation) and categorical data are presented as count (percentage).
BMI = body mass index; HDL = high-density lipoprotein; LDL = low-density lipoprotein

Supplementary Table 13. Definitions of traits and conditions analyzed in phenome-wide association analyses.

Clinical Phenotype	Definition
**Heart failure**	Self-reported history of heart failure or cardiomyopathy during verbal interview with trained nurse; **or** hospitalization with or death due to ICD-10 code for hypertensive heart disease, cardiomyopathy or heart failure (I11.0, I13.0, I13.2, I25.5, I42.0, I42.5, I42.8, I42.9, I50.0, I50.1, I50.9); **or** hospitalization with ICD-9 code for heart failure or other primary cardiomyopathies (4254, 4280, 4281, 4289); **excluding** individuals with history of hypertrophic cardiomyopathy during verbal interview with trained nurse, or hospitalization with or death due to ICD-10 code for hypertrophic cardiomyopathy (I42.1, I42.2)
**Hypertrophic cardiomyopathy**	Self-reported history of hypertrophic cardiomyopathy during a verbal interview with a trained nurse; **or** hospitalization or death due to an ICD-10 code for hypertrophic cardiomyopathy (I42.1, I42.2); **or** hospitalization with an ICD-9 code for hypertrophic cardiomyopathy (425.11, 425.18).
**Coronary artery disease**	Self-reported history of myocardial infarction (MI), coronary artery bypass grafting, coronary artery angioplasty or triple heart bypass during verbal interview with trained nurse; **or** hospitalization with or death due to ICD-10 code for acute or subsequent myocardial infarction (I21, I22, I23, I24.1, I25.2); **or** hospitalization with ICD-9 code for myocardial infarction (410, 411, 412); **or** hospitalization with OPCS-4 code for coronary artery bypass grafting (K40, K41, K45) or coronary angioplasty ± stenting (K49, K50.2, K75)
**Stroke**	Self-reported history of stroke during verbal interview with trained nurse; **or** hospitalization with or death due to ICD-10 code for nontraumatic subarachnoid hemorrhage, nontraumatic intracerebral hemorrhage, cerebral infarction, or unspecified stroke (I60-64); **or** hospitalization with or death due to ICD-9 code for subarachnoid hemorrhage, intracerebral hemorrhage, occlusion of cerebral arteries, or acute cerebrovascular disease (430, 431, 434, 436), as adjudicated centrally by the UK Biobank (http://biobank.ctsu.ox.ac.uk/crystal/refer.cgi?id=462)
**Ischemic stroke**	Self-reported history of ischemic stroke during verbal interview with trained nurse; **or** hospitalization with or death due to ICD-10 code for cerebral infarction, or unspecified stroke (I63, 64); **or** hospitalization with or death due to ICD-9 code for occlusion of cerebral arteries or acute cerebrovascular disease (434, 436), as adjudicated centrally by the UK Biobank (http://biobank.ctsu.ox.ac.uk/crystal/refer.cgi?id=462)
**Intracerebral hemorrhage**	Self-reported history of brain hemorrhage during verbal interview with trained nurse; **or** hospitalization with or death due to ICD-10 code for nontraumatic intracerebral hemorrhage (I61); **or** hospitalization with or death due to ICD-9 code for intracerebral hemorrhage (431), as adjudicated centrally by the UK Biobank (http://biobank.ctsu.ox.ac.uk/crystal/refer.cgi?id=462)
**Pulmonary hypertension**	Hospitalization with or death due to ICD-10 code for primary or other secondary pulmonary hypertension (I27.0, I27.2); **or** hospitalization with ICD-9 code for primary pulmonary hypertension (4160)
**Atrial fibrillation or flutter**	Self-reported history of atrial fibrillation, atrial flutter, or cardioversion during verbal interview with trained nurse; **or** hospitalization with or death due to ICD-10 code for atrial fibrillation or atrial flutter (I48); **or** hospitalization with ICD-9 code for atrial fibrillation or atrial flutter (4273); **or** hospitalization with OPCS-4 code for percutaneous transluminal ablation (K57.1, K 62.1, K62.2, K62.3, K62.4)
**Venous thromboembolism**	Self-reported history of venous thromboembolic disease, pulmonary embolism or deep venous thrombosis during verbal interview with trained nurse; **or** hospitalization with or death due to ICD-10 code for pulmonary embolism (I26), phlebitis or thrombophlebitis (I80.0-I80.3, I80.8-I80.9), portal vein thrombosis (I81), Budd-Chiari syndrome (I82.0), or other coagulation defects (D68); **or** hospitalization with ICD-9 code for pulmonary embolism or phlebitis/thrombophlebitis (4151, 4511); **or** hospitalization with OPCS-4 code

	for insertion of inferior vena cava filter or open thrombectomy of lower extremity vein (L79.1, L90.2)
**Peripheral vascular disease**	Self-reported history of peripheral vascular disease, arterial embolism, intermittent claudication, leg artery bypass, leg artery angioplasty, or leg amputation during verbal interview with trained nurse; **or** hospitalization with or death due to ICD-10 code for atherosclerosis of (non-coronary) arteries or peripheral vascular disease (I70.0, I70.00, I70.01, I70.2, I70.20, I70.21, I70.8, I70.80, I70.9, I70.90, I73.8 or I73.9); **or** hospitalization with ICD-9 code for atherosclerosis of arteries or peripheral vascular disease (4400, 4402, 4438, 4439); **or** hospitalization with OPCS-4 coded procedure for leg amputation, or leg artery procedure such as bypass, stent or angioplasty (X09.3-09.5, L21.6, L51.3, L51.6, L51.8, L52.1, L52.2, L54.1, L54.4, L54.8, L59.1-L59.8, L60.1, L60.2, L63.1, L63.5, L63.9, L66.7)
**Hypertension**	Self-reported history of hypertension, essential hypertension or high blood pressure during verbal interview with trained nurse; **or** hospitalization with or death due to ICD-10 code for essential hypertension, hypertensive heart disease, hypertensive renal disease, or secondary hypertension (I10, I11, I12, I13, I15); **or** hospitalization with ICD-9 code for essential hypertension, hypertensive heart disease, hypertensive renal disease, or secondary hypertension (401, 402, 403, 404, 405)
**Hypercholesterolemia**	Self-reported history of high cholesterol during verbal interview with trained nurse; **or** hospitalization with or death due to ICD-10 code for hypercholesterolemia, hypertriglyceridemia, or hyperlipidemia (E78.0-E78.2, E78.4, E78.5)
**Supraventricular arrhythmia –general inclusive definition**	Self-reported history of Wolff-Parkinson-White syndrome or supraventricular tachycardia during verbal interview with trained nurse; **or** hospitalization with or death due to ICD-10 code for Preexcitation syndrome, supraventricular tachycardia, atrial premature depolarization (I45.6, I47.1, I49.1); **or** hospitalization with ICD-9 code for anomalous atrioventricular excitation or paroxysmal supraventricular tachycardia (4267, 4270); **or** hospitalization with OPCS-4 coded procedure for open division of accessory pathway, percutaneous transluminal ablation of accessory pathway/atrial wall/conduction system (K52.4, K57.2, K57.4, K57.5)
**Bradyarrhythmia – sinus node dysfunction**	Self-reported history of sick sinus syndrome during verbal interview with trained nurse; **or** hospitalization with or death due to ICD-10 code for sick sinus syndrome (I49.5)
**Bradyarrhythmia - AV block / distal conduction disease**	Hospitalization with or death due to ICD-10 code for atrioventricular and intraventricular block (I44, I45.0-I45.5); **or** hospitalization with ICD-9 code for atrioventricular or intraventricular block (4260, 4261, 4263, 4264, 4265, 4266)
**Ventricular arrhythmia - General inclusive definition**	Hospitalization with or death due to ICD-10 code for ventricular arrhythmias, ventricular premature depolarization or cardiac arrest (I46.0, I46.1, I46.9, I47.0, I47.2, I49.0, I49.3); **or** hospitalization with ICD-9 code for ventricular arrhythmias or cardiac arrest (4271, 4274, 4275); **or** hospitalization with OPCS-4 coded procedure for percutaneous transluminal/radiofrequency ablation (K57.6, K64.1) or resuscitation/defibrillation (X50.3, X50.4, X50.8, X50.9)
**Aortic stenosis**	Self-reported history of aortic stenosis during verbal interview with trained nurse; **or** hospitalization with or death due to ICD-10 code for rheumatic aortic stenosis (I06.0, I06.2) or nonrheumatic aortic stenosis (I35.0, I35.2)
**Aortic regurgitation**	Self-reported history of aortic regurgitation during verbal interview with trained nurse; **or** hospitalization with or death due to ICD-10 code for rheumatic aortic insufficiency (I06.1, I06.2) or nonrheumatic aortic insufficiency (I35.0, I35.2); **or** hospitalization with ICD-9 code for rheumatic aortic insufficiency (3951)
**Mitral stenosis**	Self-reported history of mitral stenosis during verbal interview with trained nurse; **or** hospitalization with or death due to ICD-10 code for rheumatic and nonrheumatic mitral stenosis (I05.0, I34.2); **or** hospitalization with ICD-9 code for mitral stenosis ± insufficiency (3940, 3942); **or** hospitalization with OPCS-4 code for percutaneous transluminal mitral valvotomy (K35.1)
**Mitral regurgitation**	Self-reported history of mitral regurgitation during verbal interview with trained nurse; **or** hospitalization with or death due to ICD-10 code for rheumatic and

	nonrheumatic mitral insufficiency (I05.1, I05.2, I34.0); **or** hospitalization with ICD-9 code for mitral insufficiency (3942); **or** hospitalization with OPCS-4 code for mitral valve annuloplasty (K34.1)
**Mitral valve prolapse**	Self-reported history of mitral valve prolapse during verbal interview with trained nurse; **or** hospitalization with or death due to ICD-10 code for mitral valve prolapse (I34.1)
**Tricuspid stenosis**	Hospitalization with or death due to ICD-10 code for rheumatic and nonrheumatic tricuspid stenosis (I07.0, I07.2, I36.0)
**Tricuspid regurgitation**	Hospitalization with or death due to ICD-10 code for rheumatic and nonrheumatic tricuspid regurgitation (I07.1, I07.2, I36.1); **or** hospitalization with OPCS-4 code for tricuspid valve annuloplasty (K34.2)
**Pulmonary stenosis**	Hospitalization with or death due to ICD-10 code for pulmonary stenosis (I37.0, I37.2)
**Pulmonary regurgitation**	Hospitalization with or death due to ICD-10 code for pulmonary insufficiency (I37.1, I37.2)
**Congenital heart disease**	Hospitalization with or death due to ICD-10 code for congenital malformations of cardiac chambers/connexions, cardiac septa, aortic/mitral/tricuspid/pulmonary valves, and other/unspecified malformation (Q20.1, Q20.2, Q20.3, Q20.4, Q20.5, Q20.6, Q20.8, Q20.9; Q21, Q22, Q23, Q24); **or** hospitalization with ICD-9 code for congenital anomalies of the heart and cardiac septa (745, 746); **or** hospitalization with OPCS-4 code for repair of tetralogy of Fallot (K04), correction of total anomalous pulmonary venous connection (K07), repair of defect of atrioventricular/interatrial/interventricular/unspecified septum (K09, K10, K11, K12), transluminal repair of septal defect (K13), transluminal repair of atrial septum or patent oval foramen with prosthesis (K16.3, K16.5), creation of valved/other cardiac conduit (K18, K19, refashioning of atrium (K20)
**Diabetes mellitus, type 1**	Self-reported history of Type 1 diabetes during verbal interview with trained nurse; **or** hospitalization with or death due to ICD-10 code for insulin-dependent diabetes mellitus (E10)
**Diabetes mellitus, type 2**	Self-reported history of type 2 diabetes during verbal interview with trained nurse; **or** hospitalization with or death due to ICD-10 code for non-insulin-dependent diabetes mellitus (E11)
**Diabetes mellitus, all**	Self-reported history of diabetes, gestational diabetes, type 1/type 2 or insulin use during verbal interview with trained nurse; **or** hospitalization with or death due to ICD-10 code for insulin-dependent and non-insulin-dependent diabetes mellitus (E10, E11), malnutrition-related diabetes mellitus (E12), other specified/unspecified diabetes mellitus (E13, E14); **or** hospitalization with ICD-9 code for diabetes mellitus with/without complications (2500, 2501, 2503, 2504, 2505, 2509)
**Hyperthyroidism**	Self-reported history of hyperthyroidism/thyrotoxicosis during verbal interview with trained nurse; **or** hospitalization with or death due to ICD-10 code for hyperthyroidism/thyrotoxicosis (E05); **or** hospitalization with ICD-9 code for thyrotoxicosis with/without goiter (242)
**Hypothyroidism**	Self-reported history of hypothyroidism/myxoedema during verbal interview with trained nurse; **or** hospitalization with or death due to ICD-10 code for hypothyroidism (E03); **or** hospitalization with ICD-9 code for acquired hypothyroidism (244)
**Gout**	Self-reported history of gout during verbal interview with trained nurse; **or** hospitalization with or death due to ICD-10 code for gout (M10.0, M10.2, M10.3, M10.4, M10.9); **or** hospitalization with ICD-9 code for gout (274)
**Enlarged prostate**	Self-reported history of enlarged prostate during verbal interview with trained nurse; **or** hospitalization with or death due to ICD-10 code for hyperplasia of prostate (N40); **or** hospitalization with ICD-9 code for hyperplasia of prostate (600)
**Uterine fibroids**	Self-reported history of uterine fibroids or myomectomy/fibroid removal during verbal interview with trained nurse; **or** hospitalization with or death due to ICD-10 code for leiomyoma of uterus (D25); **or** hospitalization with ICD-9 code for uterine leiomyoma (218)

**Chronic kidney disease**	Self-reported history of kidney failure ± dialysis, kidney nephropathy, IgA nephropathy, diabetic nephropathy or kidney transplant during verbal interview with trained nurse; **or** hospitalization with or death due to ICD-10 code for hypertensive renal disease, chronic renal failure, end stage renal failure or chronic kidney disease (I12.0, I13.1, I13.2, N18, N18.0-18.5, N18.8, N18.9); **or** hospitalization with ICD-9 code due to chronic renal failure (585, 5859); **or** hospitalization with OPCS-4 coded procedure for kidney transplantation (M01.1-01.5, M01.8, M01.9)
**Gastroesophageal reflux disease**	Self-reported history of gastroesophageal/gastric reflux during verbal interview with trained nurse; **or** hospitalization with or death due to ICD-10 code for gastroesophageal reflux disease (K21); **or** hospitalization with ICD-9 code for esophageal reflux (53010, 53011)
**Irritable bowel syndrome**	Self-reported history of irritable bowel syndrome during verbal interview with trained nurse; **or** hospitalization with or death due to ICD-10 code for irritable bowel syndrome (K58); **or** hospitalization with ICD-9 code for irritable colon (5641)
**Cholelithiasis or Gallstones**	Self-reported history of cholelithiasis/gallstones or gallstone removal during verbal interview with trained nurse; **or** hospitalization with or death due to ICD-10 code for gallstone ileus (K56.3) or cholelithiasis (K80; **or** hospitalization with ICD-9 code for cholelithiasis (574); **or** hospitalization with OPCS-4 coded procedure for open removal/percutaneous dissolution/fragmentation of gall bladder calculus (J21.1, J24.2, J24.3, J26.1)
**Inflammatory bowel disease**	Self-reported history of inflammatory bowel disease, Crohn's disease, or ulcerative colitis during verbal interview with trained nurse; **or** hospitalization with or death due to ICD-10 code for Crohn's disease or ulcerative colitis (K50, K51); **or** hospitalization with ICD-9 code for regional enteritis, idiopathic proctocolitis (555, 556)
**Diverticular disease**	Self-reported history of diverticular disease during verbal interview with trained nurse; **or** hospitalization with or death due to ICD-10 code for diverticular disease of intestine (K57); **or** hospitalization with ICD-9 code for diverticula of intestine (562)
**Pancreatitis**	Self-reported history of pancreatitis during verbal interview with trained nurse; **or** hospitalization with or death due to ICD-10 code for acute pancreatitis (K85) or alcohol-induced/other chronic pancreatitis (K86.0, K86.1); **or** hospitalization with ICD-9 code for acute/chronic pancreatitis (5770, 5771)
**Migraine**	Self-reported history of migraine during verbal interview with trained nurse; **or** hospitalization with or death due to ICD-10 code for migraine (G43)
**Depression**	Self-reported history of depression during verbal interview with trained nurse; **or** hospitalization with or death due to ICD-10 code for depressive episode or recurrent depressive disorder (F32, F33); **or** hospitalization with ICD-9 code for depressive disorder (3119)
**Bipolar disorder**	Self-reported history of mania/bipolar disorder/manic depression during verbal interview with trained nurse; **or** hospitalization with or death due to ICD-10 code for bipolar affective disorder (F31)
**Anxiety**	Self-reported history of anxiety/panic attacks during verbal interview with trained nurse; **or** hospitalization with or death due to ICD-10 code for anxiety disorders (F41)
**Schizophrenia**	Self-reported history of schizophrenia during verbal interview with trained nurse; **or** hospitalization with or death due to ICD-10 code for schizophrenia (F20)
**Post-traumatic stress disorder**	Self-reported history of post-traumatic stress disorder during verbal interview with trained nurse; **or** hospitalization with or death due to ICD-10 code for post-traumatic stress disorder (F43.1)
**Multiple sclerosis**	Self-reported history of multiple sclerosis during verbal interview with trained nurse; **or** hospitalization with or death due to ICD-10 code for multiple sclerosis (G35); **or** hospitalization with ICD-9 code for multiple sclerosis (3409)
**Parkinson's disease**	Self-reported history of Parkinson's disease during verbal interview with trained nurse; **or** hospitalization with or death due to ICD-10 code for Parkinson's disease (G20) or dementia in Parkinson's disease (F02.3); **or** hospitalization with ICD-9 code for paralysis agitans (3320)

**Epilepsy**	Self-reported history of epilepsy during verbal interview with trained nurse; **or** hospitalization with or death due to ICD-10 code for epilepsy (G40); **or** hospitalization with ICD-9 code for epilepsy (3450, 3451, 3452, 3454, 3459)
**Alzheimer's / Dementia**	Self-reported history of dementia/Alzheimer's/cognitive impairment during verbal interview with trained nurse; **or** hospitalization with or death due to ICD-10 code for dementia in Alzheimer's disease (F00)
**Back pain**	Self-reported history of back pain or sciatica during verbal interview with trained nurse; **or** hospitalization with or death due to ICD-10 code for Dorsalgia (M54); **or** hospitalization with ICD-9 code for cervicalgia, spinal stenosis, pain in thoracic spine, lumbago, sciatica, thoracic or lumbosacral neuritis/radiculitis, unspecified backache (7231, 7240, 7241, 7242, 7243, 7244, 7245)
**Sciatica**	Self-reported history of sciatica during verbal interview with trained nurse; **or** hospitalization with or death due to ICD-10 code for sciatica (M54.3); **or** hospitalization with ICD-9 code for sciatica (7243)
**Osteoporosis**	Self-reported history of osteoporosis during verbal interview with trained nurse; **or** hospitalization with or death due to ICD-10 code for osteoporosis with/without pathological fracture (M80, M81); **or** hospitalization with ICD-9 code for osteoporosis (7330)
**Osteoarthritis**	Self-reported history of osteoarthritis during verbal interview with trained nurse; **or** hospitalization with or death due to ICD-10 code for polyarthrosis, coxarthrosis, gonarthrosis, first carpometacarpal or other arthrosis (M15, M16, M17, M18, M19); **or** hospitalization with ICD-9 code for osteoarthrosis (715)
**Rheumatoid arthritis**	Self-reported history of rheumatoid arthritis during verbal interview with trained nurse; **or** hospitalization with or death due to ICD-10 code for rheumatoid arthritis (M05, M06); **or** hospitalization with ICD-9 code for rheumatoid arthritis (714)
**Lupus erythematosus**	Self-reported history of systemic lupus erythematosus during verbal interview with trained nurse; **or** hospitalization with or death due to ICD-10 code for lupus erythematosus (L93) or systemic lupus erythematosus (M32.1, M32.8, M32.9); **or** hospitalization with ICD-9 code for lupus erythematosus (6954)
**Sarcoidosis**	Self-reported history of sarcoidosis during verbal interview with trained nurse; **or** hospitalization with or death due to ICD-10 code for sarcoidosis (D86); **or** hospitalization with ICD-9 code for sarcoidosis (135)
**Psoriasis**	Self-reported history of psoriasis during verbal interview with trained nurse; **or** hospitalization with or death due to ICD-10 code for psoriasis (L40), psoriatic arthropathy, arthritis mutilans, psoriatic spondylitis (M07.0, M07.1, M07.2, M07.3); **or** hospitalization with ICD-9 code for psoriasis/psoriatic arthropathy (6960, 6961)
**Dermatitis**	Self-reported history of eczema/dermatitis during verbal interview with trained nurse; **or** hospitalization with or death due to ICD-10 code for atopic dermatitis, seborrhoeic dermatitis, diaper dermatitis, allergic contact dermatitis, irritant/unspecified contact dermatitis (L20, L21, L22, L23, L24, L25, L26, L27, L30), Lichen simplex chronicus and prurigo (L28), pruritus (L29); **or** hospitalization with ICD-9 code for atopic/contact dermatitis (691, 692)
**Iron deficiency anemia**	Self-reported history of iron deficiency anemia during verbal interview with trained nurse; **or** hospitalization with or death due to ICD-10 code for iron deficiency anemia (D50); **or** hospitalization with ICD-9 code for iron deficiency anemia (280)
**Asthma**	Self-reported history of asthma during verbal interview with trained nurse; **or** hospitalization with or death due to ICD-10 code for asthma or status asthmaticus (J45, J46); **or** hospitalization with ICD-9 code for asthma (493)
**Chronic obstructive pulmonary disease**	Self-reported history of chronic obstructive airways/emphysema during verbal interview with trained nurse; **or** hospitalization with or death due to ICD-10 code for chronic bronchitis, emphysema, or other chronic obstructive pulmonary disease (J41, J42, J43, J44); **or** hospitalization with ICD-9 code for chronic bronchitis, emphysema or unspecified chronic airways obstruction (491, 492, 496)
**Pneumonia**	Self-reported history of pneumonia during verbal interview with trained nurse; **or** hospitalization with or death due to ICD-10 code for pneumonia (J12, J13,

	J14, J15, J16, J17, J18); **or** hospitalization with ICD-9 code for pneumonia (481, 482, 483, 484, 485, 486)
**Allergic rhinitis**	Self-reported history of hayfever/allergic rhinitis during verbal interview with trained nurse; **or** hospitalization with or death due to ICD-10 code for allergic rhinitis (J30.1-J30.4); **or** hospitalization with ICD-9 code for allergic rhinitis (477)
**Sleep apnea**	Self-reported history of sleep apnea during verbal interview with trained nurse; **or** hospitalization with or death due to ICD-10 code for sleep apnea (G47.3)
**Cataract**	Self-reported history of cataract during verbal interview with trained nurse; **or** hospitalization with or death due to ICD-10 code for cataract (H25, H26); **or** hospitalization with ICD-9 code for cataract (366)
**Glaucoma**	Self-reported history of glaucoma during verbal interview with trained nurse; **or** hospitalization with or death due to ICD-10 code for glaucoma (H40); **or** hospitalization with ICD-9 code for glaucoma (365)
**Lung cancer**	Self-reported history of lung cancer during verbal interview with trained nurse; **or** hospitalization with or death due to ICD-10 code for malignant neoplasm of bronchus and lung (C34); **or** hospitalization with ICD-9 code for malignant neoplasm of bronchus and lung (1629)
**Breast cancer**	Self-reported history of breast cancer during verbal interview with trained nurse; **or** hospitalization with or death due to ICD-10 code for malignant neoplasm of breast (C50); **or** hospitalization with ICD-9 code for malignant neoplasm of breast (174)
**Colorectal cancer**	Self-reported history of colorectal/sigmoid/rectal cancer during verbal interview with trained nurse; **or** hospitalization with or death due to ICD-10 code for malignant neoplasm of colon (C18.0, C18.2-18.9); **or** hospitalization with ICD-9 code for malignant neoplasm of colon/rectum (1532, 1533, 1541)
**Skin cancer**	Self-reported history of skin, malignant melanoma, non-melanoma skin cancer, or squamous cell carcinoma during verbal interview with trained nurse; **or** hospitalization with or death due to ICD-10 code for malignant melanoma or other malignant neoplasms of skin (C43, C44); **or** hospitalization with ICD-9 code for malignant melanoma/neoplasm of skin (172, 173)
**Prostate cancer**	Self-reported history of prostate cancer during verbal interview with trained nurse; **or** hospitalization with or death due to ICD-10 code for malignant neoplasm of prostate (C61)
**Cervical cancer**	Self-reported history of cervical cancer during verbal interview with trained nurse; **or** hospitalization with or death due to ICD-10 code for malignant neoplasm of cervix uteri (C53)
**Bladder cancer**	Self-reported history of bladder cancer during verbal interview with trained nurse; **or** hospitalization with or death due to ICD-10 code for malignant neoplasm of bladder (C67); **or** hospitalization with ICD-9 code for malignant neoplasm of bladder (188)
**Gestational hypertension – preeclampsia**	Self-reported history of gestational hypertension/pre-eclampsia during verbal interview with trained nurse; **or** hospitalization with or death due to ICD-10 code for pre-existing hypertensive disorder with superimposed proteinuria (O11), gestational hypertension (O13, O14), eclampsia (O15); **or** hospitalization with ICD-9 code for transient hypertension of pregnancy, preeclampsia or eclampsia (6423, 6424, 6425, 6426, 6427)
**Death**	Date of death 40000 (http://biobank.ndph.ox.ac.uk/showcase/field.cgi?id=40000)
**Ever Smoked**	20160 (http://biobank.ndph.ox.ac.uk/showcase/field.cgi?id=20160)
**BMI**	23104(Impedence Measure) http://biobank.ndph.ox.ac.uk/showcase/field.cgi?id=23104
**Percentage Body Fat**	23099 (http://biobank.ndph.ox.ac.uk/showcase/field.cgi?id=23099)

BMI = body-mass index; ICD = International Classification of Disease; OPCS = Office of Population, Census and Surveys: Classification of Interventions and Procedures

Supplementary Table 14. Phenome-wide association results for rs5942634-T in the UK Biobank attaining at least nominal association (P < 0.05)

Phenotype	beta	SE	P
Hypercholesterolemia	-0.0602	5.20×10^{-03}	5.88×10^{-31}
BMI	0.0657	9.27×10^{-03}	1.39×10^{-12}
Percentage body fat	0.0674	0.0124	4.92×10^{-08}
Glaucoma	-0.0705	0.0149	2.25×10^{-06}
Ever smoked	0.0177	3.95×10^{-03}	7.45×10^{-06}
Hypertension	-0.0188	4.28×10^{-03}	1.14×10^{-05}
Diabetes mellitus, All	-0.0306	7.72×10^{-03}	7.42×10^{-05}
Diabetes mellitus, type 2	-0.0346	8.91×10^{-03}	1.02×10^{-04}
Enlarged prostate	-0.0340	9.41×10^{-03}	3.04×10^{-04}
Coronary artery disease	-0.0256	8.52×10^{-03}	2.63×10^{-03}
Gout	-0.0350	0.0129	6.52×10^{-03}
Sciatica	0.0319	0.0158	0.0431
Intracerebral hemorrhage	-0.0844	0.0420	0.0448

Logistic regression association analyses were adjusted for age, sex, array type, and principal components. P-values presented are two-sided, and p-values < 6.3×10^{-4} were considered significant given multiple-hypothesis testing.
BMI = body-mass index (kg/m^2); se = standard error.

Supplementary Table 15. Phenome-wide association results for rs5942648-A in the UK Biobank attaining at least nominal association (P < 0.05)

Phenotype	beta	se	P
Hypercholesterolemia	-0.0612	$5.20x10^{-03}$	$5.57 x10^{-32}$
BMI	0.0682	$9.26x10^{-03}$	$1.79 x10^{-13}$
Percentage body fat	0.0717	0.0123	$6.35 x10^{-09}$
Glaucoma	-0.0674	0.0149	$6.05 x10^{-06}$
Hypertension	-0.0187	$4.28x10^{-03}$	$1.17 x10^{-05}$
Ever smoked	0.0166	$3.94x10^{-03}$	$2.55 x10^{-05}$
Diabetes mellitus, type 2	-0.0357	$8.9x10^{-03}$	$6.15 x10^{-05}$
Diabetes mellitus, all	-0.0308	$7.71x10^{-03}$	$6.55 x10^{-05}$
Enlarged prostate	-0.0334	$9.4x10^{-03}$	$3.79 x10^{-04}$
Coronary artery disease	-0.0276	$8.51x10^{-03}$	$1.18 x10^{-03}$
Gout	-0.0362	0.0129	$4.89 x10^{-03}$
Intracerebral hemorrhage	-0.0896	0.0421	0.0331
Irritable bowel syndrome	-0.0244	0.0116	0.0353

Logistic regression association analyses were adjusted for age, sex, array type, and principal components. P-values presented are two-sided, and p-values < $6.3x10^{-4}$ were considered significant given multiple-hypothesis testing.
BMI = body-mass index (kg/m^2); se = standard error.

Supplementary Table 16. Phenome-wide association results for rs5985504-T in the UK Biobank attaining at least nominal association (P < 0.05)

Phenotypes	beta	se	P
Hypercholesterolemia	-0.0581	5.19 $x10^{-03}$	4.49 $x10^{-29}$
BMI	0.0742	9.25 $x10^{-03}$	1.05 $x10^{-15}$
Percentage body fat	0.0819	0.0123	3.06 $x10^{-11}$
Glaucoma	-0.0677	0.0149	5.19 $x10^{-06}$
Hypertension	-0.0176	4.27 $x10^{-03}$	3.91 $x10^{-05}$
Diabetes mellitus, type 2	-0.0358	8.89 $x10^{-03}$	5.68 $x10^{-05}$
Ever smoked	0.0155	3.94 $x10^{-03}$	8.49 $x10^{-05}$
Diabetes mellitus, all	-0.0299	7.7 $x10^{-03}$	1.05 $x10^{-04}$
Enlarged prostate	-0.0364	9.39 $x10^{-03}$	1.06 $x10^{-04}$
Coronary artery disease	-0.0275	8.5 $x10^{-03}$	1.20 $x10^{-03}$
Gout	-0.0361	0.0128	4.98 $x10^{-03}$
Sciatica	0.0383	0.0157	0.0149
Intracerebral hemorrhage	-0.0974	0.0421	0.0206
Irritable bowel syndrome	-0.0232	0.0116	0.0450

Logistic regression association analyses were adjusted for age, sex, array type, and principal components. P-values presented are two-sided, and p-values < $6.3x10^{-4}$ were considered significant given multiple-hypothesis testing.
BMI = body-mass index (kg/m^2); se = standard error.

Supplementary Table 17. Association of NMR lipoproteins with rs5942634, rs5985504, and rs5942648 in up to 6,356 FHS and MESA individuals.

SNP	Lipoprotein	Beta	SE	P
rs5942634	Very Large TRLP	0.003	0.014	0.805
rs5942634	Large TRLP	-0.014	0.016	0.366
rs5942634	Medium TRLP	-0.023	0.016	0.143
rs5942634	Small TRLP	-0.002	0.016	0.911
rs5942634	Very Small TRLP	-0.020	0.016	0.214
rs5942634	Large LDLP	0.021	0.016	0.176
rs5942634	Medium LDLP	-0.007	0.016	0.664
rs5942634	Small LDLP	-0.031	0.016	0.049
rs5942634	Large HDLP	-0.004	0.016	0.794
rs5942634	Medium HDLP	0.047	0.016	0.003
rs5942634	Small HDLP	-0.026	0.016	0.102
rs5942634	TRL Size	-0.008	0.016	0.629
rs5942634	LDL Size	0.042	0.016	0.008
rs5942634	HDL Size	0.015	0.016	0.345
rs5942634	TRL Triglycerides	-0.022	0.016	0.162
rs5942634	TRL Cholesterol	-0.015	0.016	0.351
rs5942648	Very Large TRLP	0.000	0.014	0.992
rs5942648	Large TRLP	-0.017	0.016	0.285
rs5942648	Medium TRLP	-0.008	0.016	0.615
rs5942648	Small TRLP	0.004	0.016	0.798
rs5942648	Very Small TRLP	-0.024	0.016	0.132
rs5942648	Large LDLP	0.027	0.016	0.088
rs5942648	Medium LDLP	-0.005	0.016	0.751
rs5942648	Small LDLP	-0.026	0.016	0.103
rs5942648	Large HDLP	-0.006	0.016	0.715
rs5942648	Medium HDLP	0.051	0.016	0.001
rs5942648	Small HDLP	-0.027	0.016	0.087
rs5942648	TRL Size	-0.016	0.016	0.317
rs5942648	LDL Size	0.042	0.016	0.008
rs5942648	HDL Size	0.014	0.016	0.380
rs5942648	TRL Triglycerides	-0.015	0.016	0.342
rs5942648	TRL Cholesterol	-0.011	0.016	0.496
rs5985504	Very Large TRLP	-0.007	0.014	0.624
rs5985504	Large TRLP	-0.020	0.016	0.193
rs5985504	Medium TRLP	-0.007	0.016	0.639
rs5985504	Small TRLP	0.007	0.016	0.648
rs5985504	Very Small TRLP	-0.019	0.016	0.227
rs5985504	Large LDLP	0.021	0.016	0.191
rs5985504	Medium LDLP	-0.004	0.016	0.819
rs5985504	Small LDLP	-0.022	0.016	0.159
rs5985504	Large HDLP	-0.010	0.016	0.543
rs5985504	Medium HDLP	0.043	0.016	0.007
rs5985504	Small HDLP	-0.023	0.016	0.156

rs5985504	TRL Size	-0.023	0.016	0.144
rs5985504	LDL Size	0.034	0.016	0.031
rs5985504	HDL Size	0.008	0.016	0.624
rs5985504	TRL Triglycerides	-0.019	0.016	0.236
rs5985504	TRL Cholesterol	-0.018	0.016	0.254

Linear regression association analyses were adjusted for age, sex, and lipid-lowering therapy. P-values presented are two-sided and p-values < 0.001 are considered significant accounting for multiple-hypothesis testing.

Supplementary Table 18. Association of rs5942634-T with body composition measures in the UK Biobank.

Location	Beta (kg)	SE	P
**Whole body**	0.118	0.018	4.8×10^{-11}
**Leg, right**	0.021	2.9×10^{-03}	3.6×10^{-12}
**Leg, left**	0.020	3.0×10^{-03}	3.4×10^{-12}
**Arm, right**	6.8×10^{-03}	1.2×10^{-03}	4.1×10^{-09}
**Arm, left**	7.6×10^{-03}	1.3×10^{-03}	1.7×10^{-09}
**Trunk**	0.063	0.010	4.0×10^{-10}

Linear regression association analyses were adjusted for age, sex, array type, and principal components. P-values presented are two-sided, and p-values $< 5.6 \times 10^{-3}$ were considered significant given multiple-hypothesis testing.

Supplementary Table 19. **Association of lipid-associated chrXq23 variants with visual acuity in the UK Biobank**

Variant	Beta	SE	*P*
rs5942634-A	$-1.84x10^{-4}$	$5.33x10^{-4}$	0.73
rs5942648-T	$-8.29x10^{-5}$	$5.28x10^{-4}$	0.88
rs5985504-T	$-1.04x10^{-4}$	$5.30x10^{-4}$	0.84

The effect estimates are represented in “logMAR” units, where, for example, 0 represents 20/20 vision and 1 represents 20/200 vision. Negative values represent vision better than 20/20 (e.g., -0.1 represents 20/16 vision). Linear regression association analyses were adjusted for age, sex, array type, and principal components. P-values presented are two-sided, and further adjustment was not performed for this secondary analysis.

Supplemental Table 20. Association between rs5942634 and gene expression of genes in ChrXq23 region in GTEx.

Tissue	GeneID	Gene Name	Alt Allele	Ref Allele	Beta	SE	P
Muscle Skeletal	ENSG00000101938.10	*CHRDL1*	T	A	-0.166	0.024	$1.19x10^{-11}$
Adipose Subcutaneous	ENSG00000101938.10	*CHRDL1*	T	A	-0.160	0.029	$8.64x10^{-8}$
Adipose Visceral Omentum	ENSG00000101938.10	*CHRDL1*	T	A	-0.169	0.036	$4.33x10^{-6}$
Muscle Skeletal	ENSG00000243978.4	*RTL9*	T	A	0.175	0.041	$2.66x10^{-5}$
Liver	ENSG00000101938.10	*CHRDL1*	T	A	-0.254	0.061	$5.93x10^{-5}$
Small Intestine Terminal Ileum	ENSG00000101938.10	*CHRDL1*	T	A	-0.107	0.049	0.030
Adipose Visceral Omentum	ENSG00000243978.4	*RTL9*	T	A	0.098	0.054	0.070
Muscle Skeletal	ENSG00000077264.10	*PAK3*	T	A	-0.040	0.034	0.242
Liver	ENSG00000243978.4	*RTL9*	T	A	0.070	0.068	0.305
Adipose Visceral Omentum	ENSG00000101935.5	*AMMECR1*	T	A	0.027	0.027	0.313
Small Intestine Terminal Ileum	ENSG00000068366.15	*ACSL4*	T	A	-0.037	0.037	0.320
Adipose Subcutaneous	ENSG00000077264.10	*PAK3*	T	A	-0.024	0.025	0.341
Small Intestine Terminal Ileum	ENSG00000077264.10	*PAK3*	T	A	0.053	0.056	0.353
Muscle Skeletal	ENSG00000157600.7	*TMEM164*	T	A	0.030	0.035	0.391
Muscle Skeletal	ENSG00000077274.7	*CAPN6*	T	A	0.019	0.024	0.416
Adipose Subcutaneous	ENSG00000101935.5	*AMMECR1*	T	A	0.024	0.034	0.479
Muscle Skeletal	ENSG00000068366.15	*ACSL4*	T	A	-0.015	0.022	0.489
Liver	ENSG00000101935.5	*AMMECR1*	T	A	0.030	0.060	0.621
Small Intestine Terminal Ileum	ENSG00000157600.7	*TMEM164*	T	A	0.012	0.025	0.624
Adipose Subcutaneous	ENSG00000077274.7	*CAPN6*	T	A	0.017	0.034	0.624
Liver	ENSG00000077264.10	*PAK3*	T	A	0.038	0.078	0.628
Whole Blood	ENSG00000068366.15	*ACSL4*	T	A	-0.006	0.012	0.644
Whole Blood	ENSG00000101935.5	*AMMECR1*	T	A	0.014	0.033	0.673
Adipose Subcutaneous	ENSG00000243978.4	*RTL9*	T	A	-0.016	0.039	0.682
Adipose Visceral Omentum	ENSG00000157600.7	*TMEM164*	T	A	0.010	0.025	0.695
Small Intestine Terminal Ileum	ENSG00000077274.7	*CAPN6*	T	A	0.024	0.062	0.705
Whole Blood	ENSG00000101938.10	*CHRDL1*	T	A	-0.015	0.040	0.712
Adipose Subcutaneous	ENSG00000157600.7	*TMEM164*	T	A	-0.009	0.024	0.728
Adipose Visceral Omentum	ENSG00000077274.7	*CAPN6*	T	A	-0.010	0.032	0.756
Adipose Subcutaneous	ENSG00000077279.12	*DCX*	T	A	0.010	0.035	0.785

Liver	ENSG00000068366.15	*ACSL4*	T	A	-0.018	0.071	0.797
Adipose Visceral Omentum	ENSG00000068366.15	*ACSL4*	T	A	0.005	0.024	0.852
Whole Blood	ENSG00000157600.7	*TMEM164*	T	A	-0.005	0.025	0.853
Small Intestine Terminal Ileum	ENSG00000101935.5	*AMMECR1*	T	A	-0.010	0.055	0.861
Liver	ENSG00000077274.7	*CAPN6*	T	A	0.011	0.072	0.878
Muscle Skeletal	ENSG00000101935.5	*AMMECR1*	T	A	-0.005	0.036	0.885
Adipose Visceral Omentum	ENSG00000077264.10	*PAK3*	T	A	0.005	0.036	0.885
Liver	ENSG00000157600.7	*TMEM164*	T	A	0.007	0.054	0.893
Adipose Subcutaneous	ENSG00000068366.15	*ACSL4*	T	A	0.002	0.025	0.928

Queries of lipid-associated alleles with gene expression from linear regression in the GTEx dataset are depicted. No further adjustment of effects or two-sided p-values were performed.

Supplemental Table 21. Colocalization of lipid with gene expression near index association across tissue types.

Gene	Tissue	# of SNPs in model	Effect estimate	SE	Z	P
ACSL4	Adipose_Subcutaneous	264	0.084	0.032	2.613	0.009
ACSL4	Adipose_Visceral_Omentum	264	-0.021	0.029	-0.699	0.484
ACSL4	Liver	262	-0.059	0.022	-2.606	0.009
ACSL4	Muscle_Skeletal	264	0.035	0.020	1.766	0.077
ACSL4	Small_Intestine_Terminal_Ileum	260	0.147	0.024	6.057	1.39×10^{-9}
ACSL4	Whole_Blood	264	0.058	0.033	1.761	0.078
AMMECR1	Adipose_Subcutaneous	493	0.055	0.030	1.800	0.072
AMMECR1	Adipose_Visceral_Omentum	493	-0.060	0.026	-2.297	0.022
AMMECR1	Liver	475	-0.047	0.028	-1.685	0.092
AMMECR1	Muscle_Skeletal	493	0.019	0.024	0.766	0.444
AMMECR1	Small_Intestine_Terminal_Ileum	479	-0.024	0.033	-0.732	0.464
AMMECR1	Whole_Blood	493	0.046	0.031	1.498	0.134
CAPN6	Adipose_Subcutaneous	318	0.018	0.021	0.876	0.381
CAPN6	Adipose_Visceral_Omentum	318	0.016	0.022	0.723	0.470
CAPN6	Liver	315	-0.006	0.018	-0.346	0.729
CAPN6	Muscle_Skeletal	318	0.018	0.023	0.794	0.427
CAPN6	Small_Intestine_Terminal_Ileum	314	-0.010	0.016	-0.625	0.532
CHRDL1	Adipose_Subcutaneous	674	1.104	0.011	98.007	1.35×10^{-2088}
CHRDL1	Adipose_Visceral_Omentum	674	1.295	0.028	45.956	4.31×10^{-461}
CHRDL1	Liver	658	1.439	0.019	76.238	8.12×10^{-1265}
CHRDL1	Muscle_Skeletal	674	0.933	0.013	70.439	4.41×10^{-1080}
CHRDL1	Small_Intestine_Terminal_Ileum	599	1.789	0.046	38.952	6.96×10^{-332}
CHRDL1	Whole_Blood	674	0.718	0.129	5.590	2.27×10^{-8}
DCX	Adipose_Subcutaneous	252	0.085	0.034	2.535	0.011
PAK3	Adipose_Subcutaneous	762	-0.062	0.021	-2.913	0.004
PAK3	Adipose_Visceral_Omentum	762	0.072	0.012	6.131	8.73×10^{-10}
PAK3	Liver	753	-0.071	0.016	-4.356	1.32×10^{-5}
PAK3	Muscle_Skeletal	762	0.035	0.015	2.253	0.024
PAK3	Small_Intestine_Terminal_Ileum	753	-0.022	0.016	-1.366	0.172
RTL9	Adipose_Subcutaneous	332	-0.020	0.034	-0.602	0.547
RTL9	Adipose_Visceral_Omentum	332	-0.057	0.036	-1.579	0.114
RTL9	Liver	324	-0.004	0.038	-0.107	0.915
RTL9	Muscle_Skeletal	332	-0.147	0.036	-4.129	3.64×10^{-5}
TMEM164	Adipose_Subcutaneous	465	-0.174	0.021	-8.222	2.01×10^{-16}
TMEM164	Adipose_Visceral_Omentum	465	-0.185	0.023	-8.040	8.99×10^{-16}
TMEM164	Liver	452	0.004	0.020	0.184	0.854
TMEM164	Muscle_Skeletal	465	0.099	0.029	3.459	0.001
TMEM164	Small_Intestine_Terminal_Ileum	459	-0.114	0.033	-3.485	4.93×10^{-4}
TMEM164	Whole_Blood	465	0.015	0.003	4.652	3.28×10^{-6}

Lipid effects were adjusted for age, age^2, sex, batch, and principal components of ancestry, as well as site-specific covariates where appropriate using linear regression. Gene expression effects were obtained directly from GTEx. Two-sided p-values < 0.001 were considered significant accounting for multiple-hypothesis testing.

Supplemental Table 22. Top gene-based association results on the X chromosome in TOPMed with p < 0.001.

Trait	Gene	Variant Set	# sites	# alt alleles	# sample with alt allele	P
TG	*TIMP1*	HC LOF + metaSVM	26	234	196	0.00019
TG	*HDX*	HC LOF + metaSVM	9	11	10	0.00066
TG	*SLC6A14*	HC LOF	6	8	7	0.00067
TG	*DMD*	HC LOF + metaSVM	113	669	544	0.00100

Rare variant SKAT association tests were adjusted for age, age^2, sex, batch, and principal components, as well as site-specific covariates where appropriate. Two-sided p-values < 6.7×10^{-5} were considered significant given multiple-hypothesis testing.
HC LOF = high-confidence loss-of-function; TG = triglycerides

Supplementary Figure 1. Study schematic.

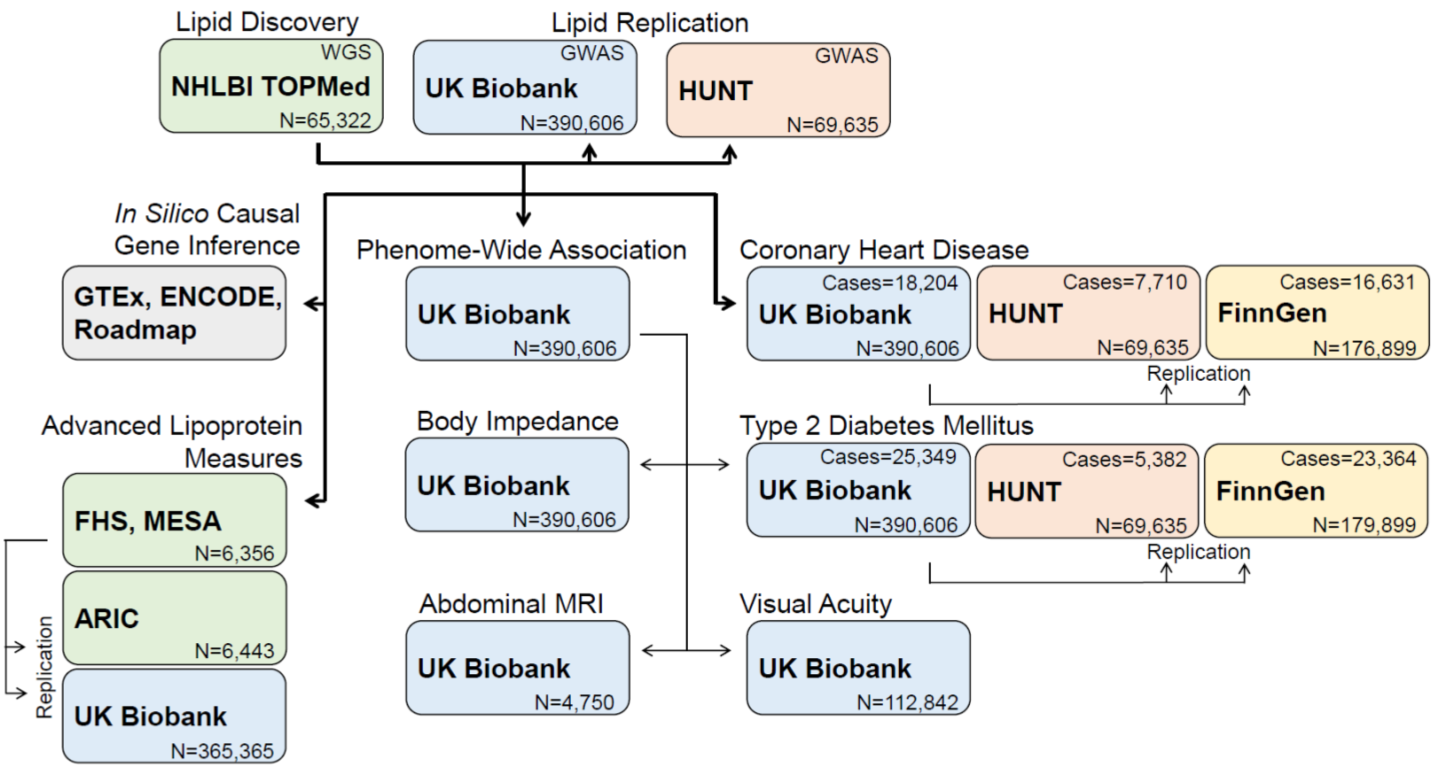

Flow chart of the overall study, including cohorts and outcomes, is shown.

Supplementary Figure 2. Principal component plots of TOPMed participants.

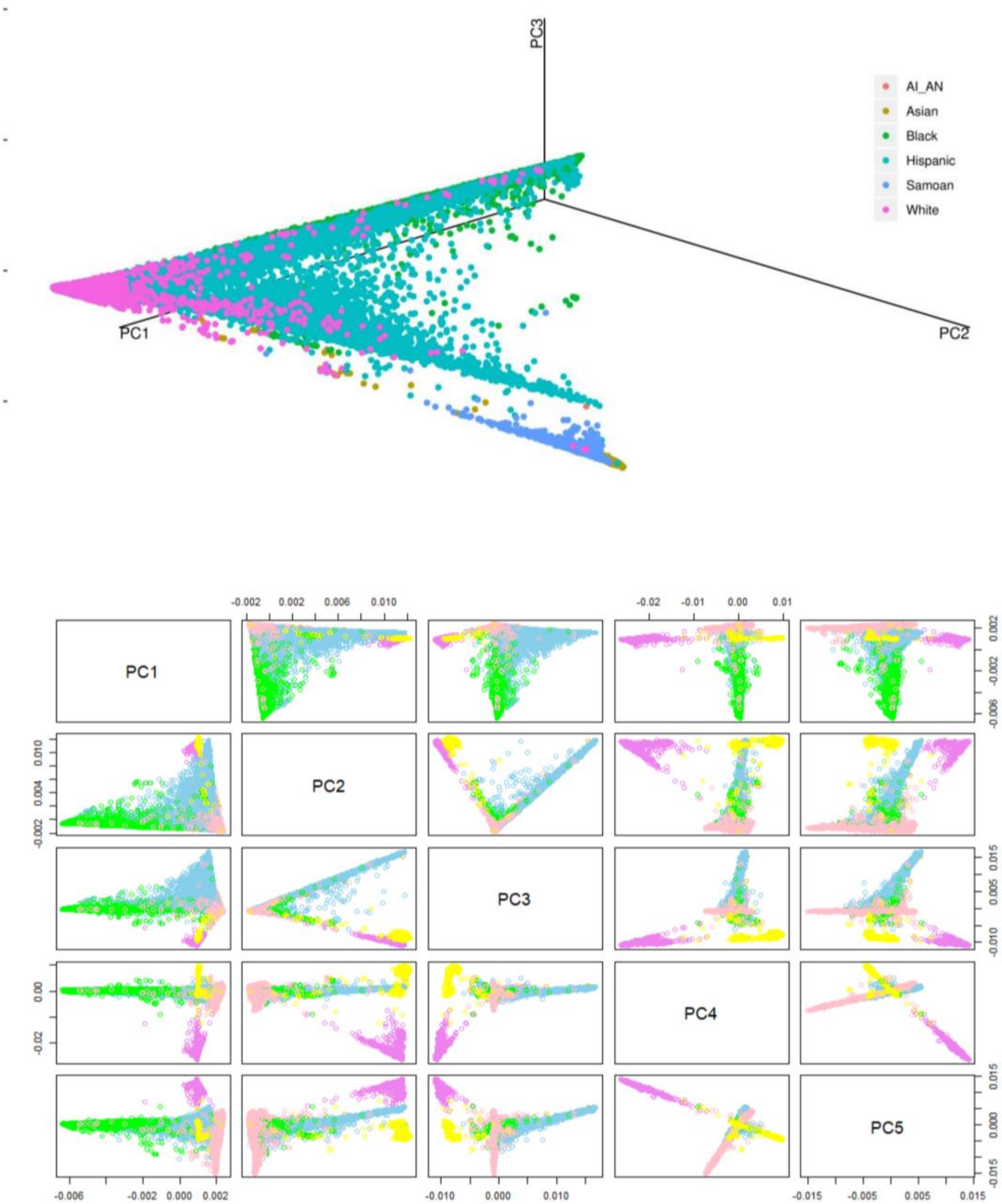

Principal components of ancestry of TOPMed participants are depicted colored by self-identified race. AI_AN = American Indian / Native American / Alaskan Native; PC = principal components

Supplementary Figure 3. Distributions of blood lipid levels by TOPMed cohort.

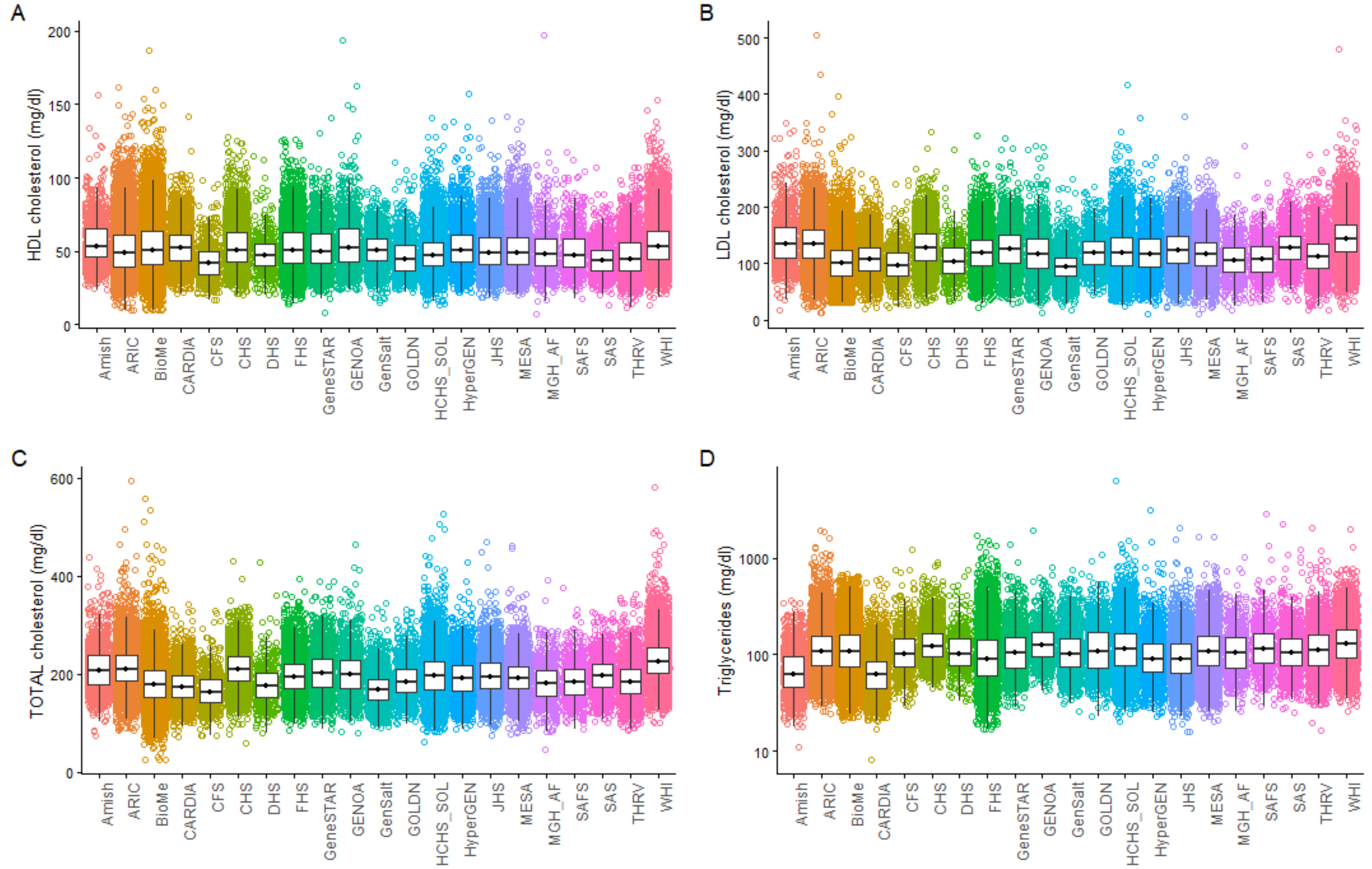

(A) HDL cholesterol, (B) LDL cholesterol, (C) total cholesterol, and (D) triglycerides. Triglycerides are displayed on a $\log 10$ scale. Sample sizes for each cohort are listed in Supplementary Table 4. For the boxplots, the center diamond is the mean, center line is the median, top box bound is the third quartile, top whisker end is the third quartile plus 1.5 times the interquartile range, bottom box bound is the first quartile, and bottom whisker end is the first quartile minus 1.5 times the interquartile range. HDL = high-density lipoprotein; LDL = low-density lipoprotein.

Supplementary Figure 4. Normalized adjusted residuals used for association analyses in TOPMed.

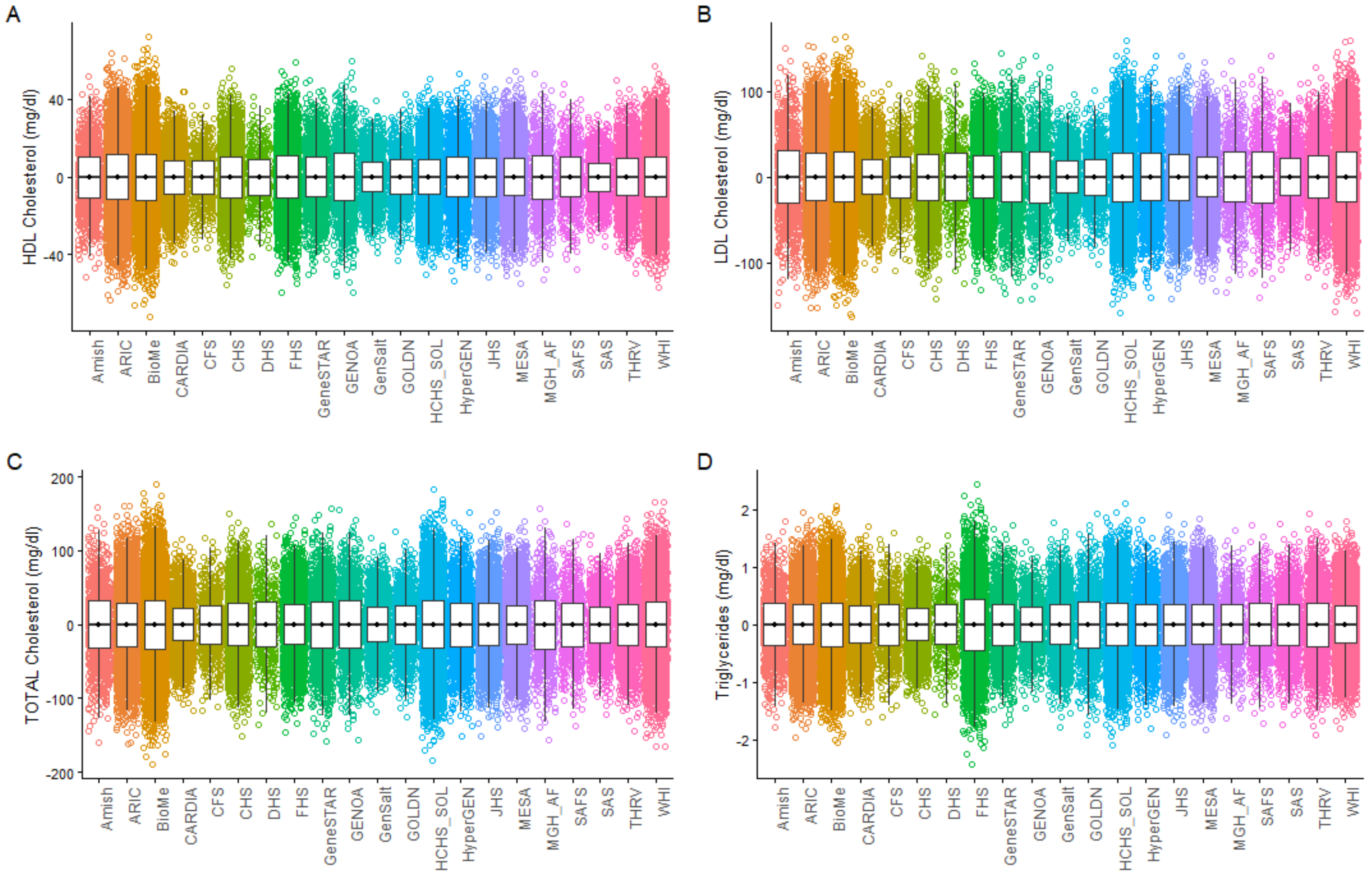

(A) HDL cholesterol, (B) LDL cholesterol, (C) total cholesterol, and (D) triglycerides. Sample sizes for each cohort are listed in Supplementary Table 4. For the boxplots, the center diamond is the mean, center line is the median, top box bound is the third quartile, top whisker end is the third quartile plus 1.5 times the interquartile range, bottom box bound is the first quartile, and bottom whisker end is the first quartile minus 1.5 times the interquartile range.
HDL = high-density lipoprotein; LDL = low-density lipoprotein.

Supplementary Figure 5. Quantile-quantile plots of chromosome X genetic variants' associations with lipid levels in TOPMed.

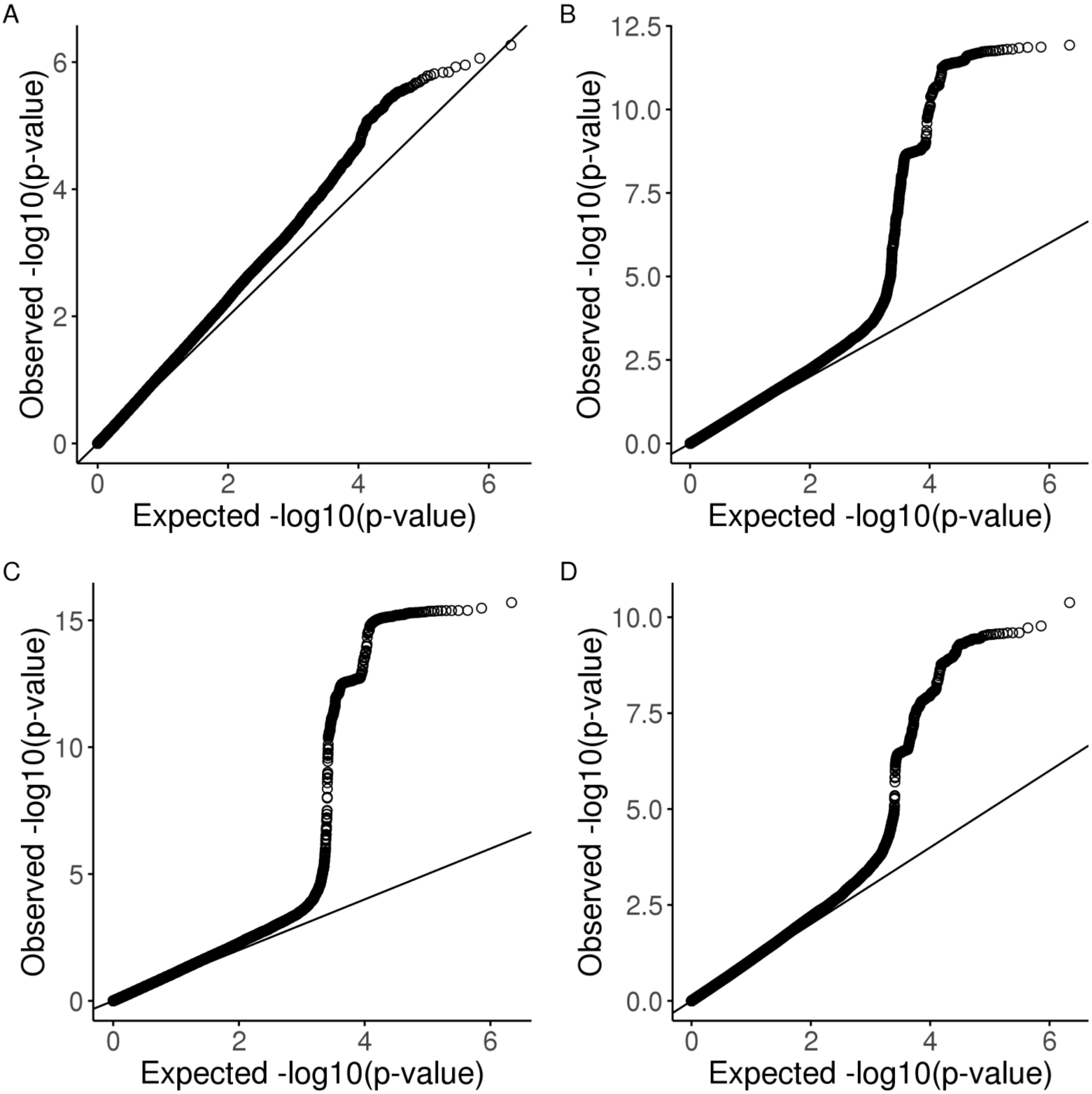

(A) HDL cholesterol (λ=1.11), (B) LDL cholesterol (λ=1.13), (C) total cholesterol (λ=1.17), and (D) triglycerides (λ=1.09).
HDL = high-density lipoprotein; LDL = low-density lipoprotein.

Supplementary Figure 6. Manhattan plots of chromosome X genetic variants' associations with lipid levels in TOPMed.

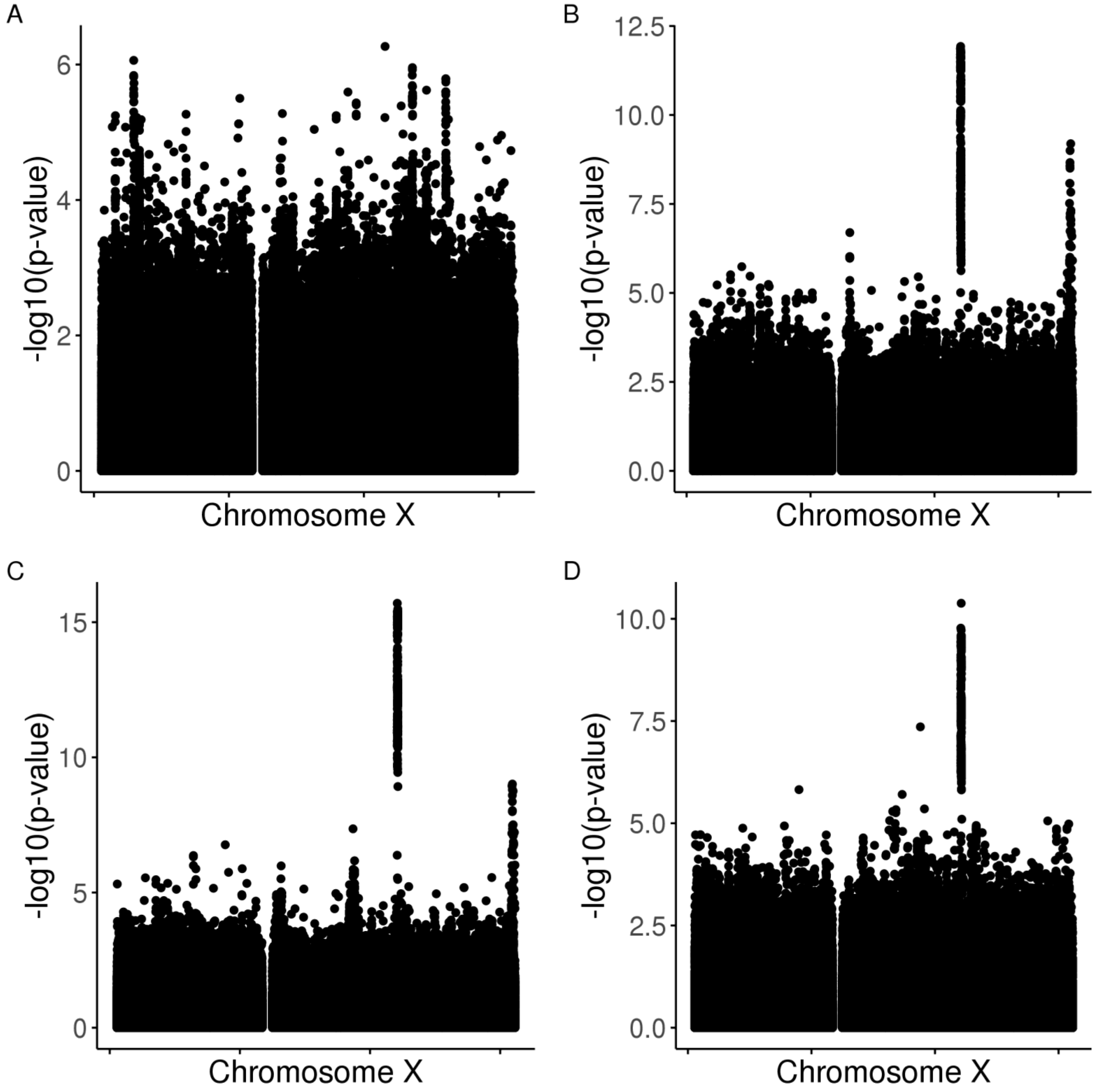

(A) HDL cholesterol, (B) LDL cholesterol, (C) total cholesterol, and (D) triglycerides.
HDL = high-density lipoprotein; LDL = low-density lipoprotein.

Supplementary Figure 7. LocusZoom plots for associated variants at chrXq23 with total cholesterol in TOPMed.

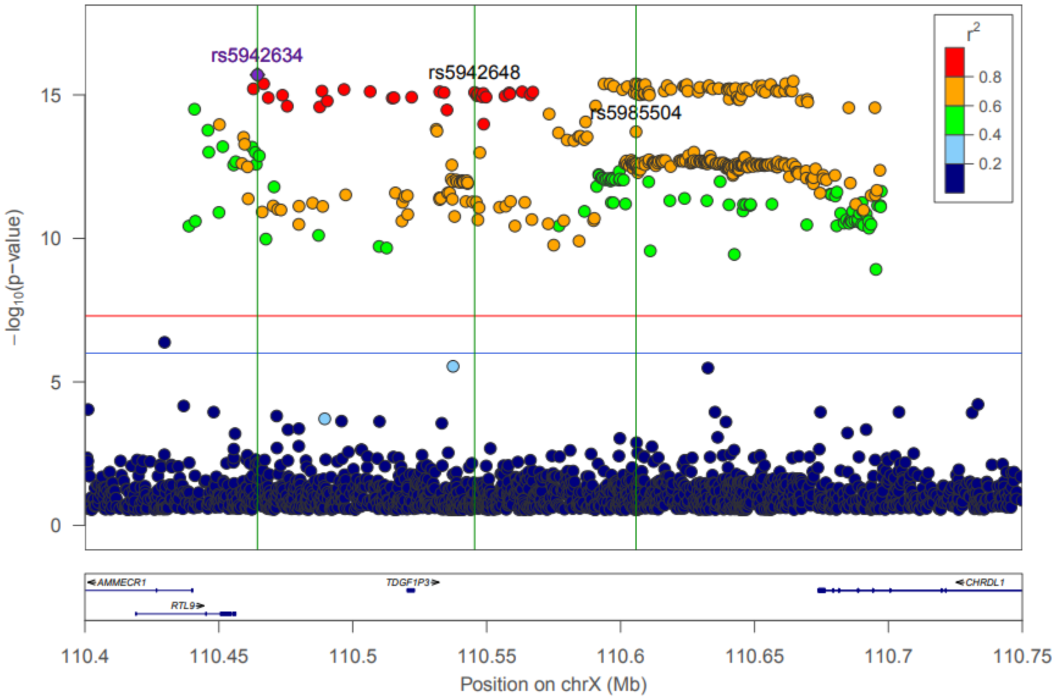

Regional association plot at chromosome Xq23 in TOPMed for the association of total cholesterol. Lead lipid-associated variants in Table 1 are indicated.

Supplementary Figure 8. ChrXq23 variants associated with total cholesterol and predicted liver enhancer regions.

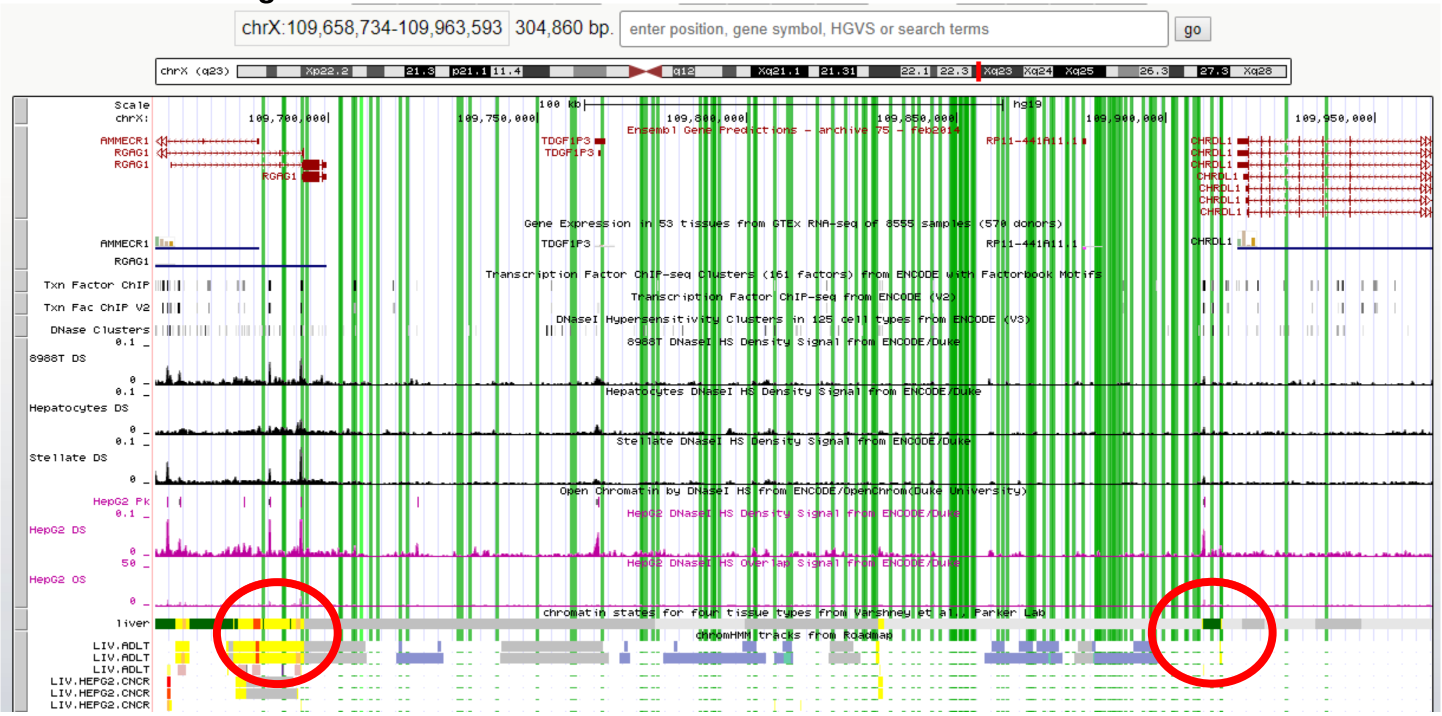

Yellow regions are predicted adult liver enhancers. Green vertical lines represent chrXq23 variants associated with total cholesterol with two-sided P < $1x10^{-13}$ (N = 119 variants) after adjustment for age, age^2, sex, batch, and principal components of ancestry, as well as site-specific covariates where appropriate using linear regression. In this model, accounting for multiple-hypothesis testing, p-values < $5.7x10^{-9}$ are considered significant.

Supplementary Figure 9. Proposed enhancer contacts combining reference Hi-C, ChIA-PET, DHS-linkage, and gene expression data

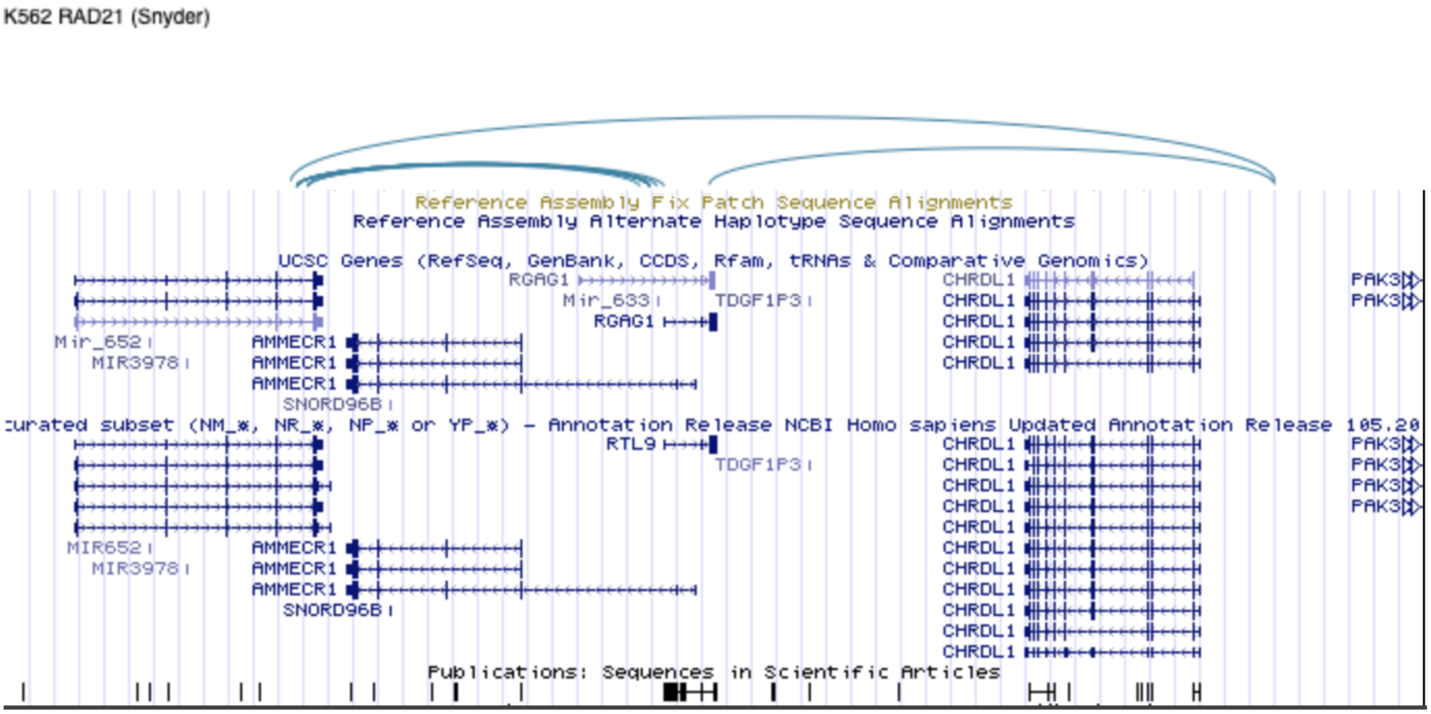

Contacts to rs5942634 (central contacts) are depicted using virtual 4C data (http://promoter.bx.psu.edu/hi-c/virtual4c.php) using K562 cell line data.[42] A contact upstream of *CHRDL1* is depicted.

Supplementary Figure 10. Correlating chrXq23 cholesterol effects with cardiometabolic outcome effects.

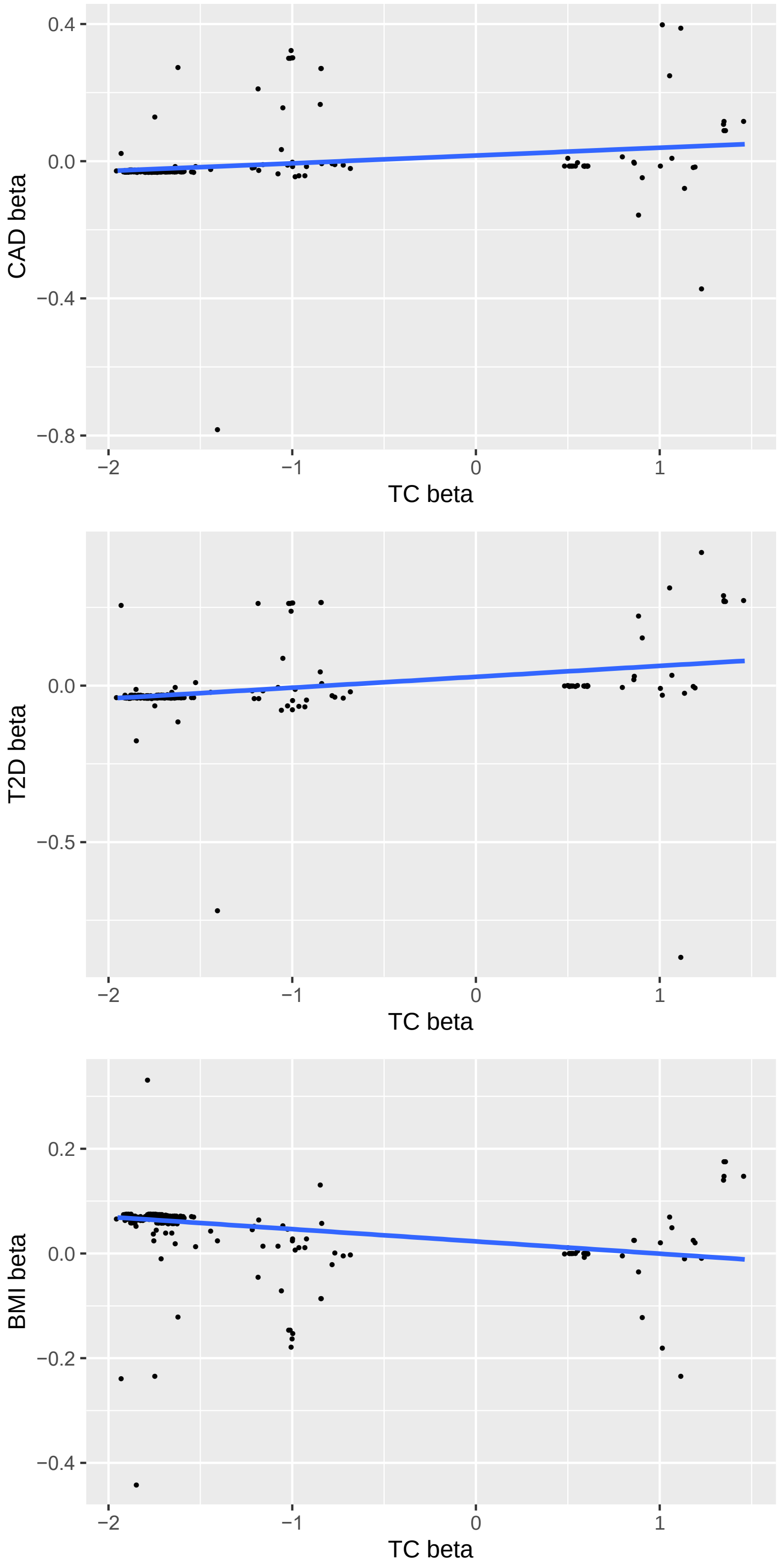

Effects of ChrXq23 variants on total cholesterol (TC) vs effects of these variants on coronary artery disease (CAD), type 2 diabetes (T2D), and BMI.

Supplementary Figure 11. Correlation between total cholesterol and gene expression variant effects at chrXq23.

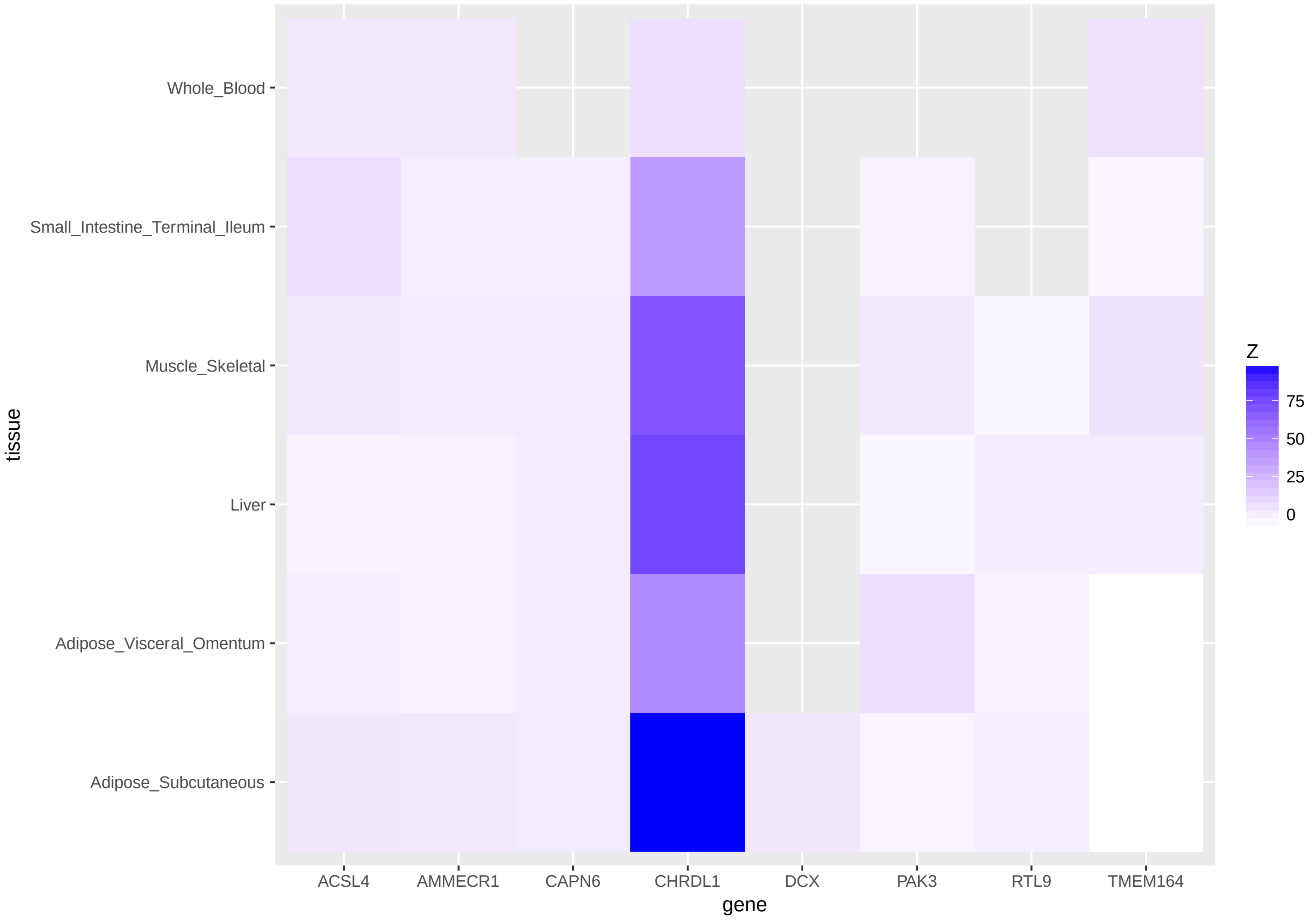

Correlation of Z-scores (beta/standard error) between total cholesterol ~ variant and gene expression ~ variant.

REFERENCES

1. The Atherosclerosis Risk in Communities (ARIC) Study: design and objectives. The ARIC investigators. *American journal of epidemiology* **129**, 687-702 (1989).

2. Mitchell BD*, et al.* The genetic response to short-term interventions affecting cardiovascular function: rationale and design of the Heredity and Phenotype Intervention (HAPI) Heart Study. *American heart journal* **155**, 823-828 (2008).

3. Nadkarni GN*, et al.* Apolipoprotein L1 Variants and Blood Pressure Traits in African Americans. *Journal of the American College of Cardiology* **69**, 1564-1574 (2017).

4. Hughes GH*, et al.* Recruitment in the Coronary Artery Disease Risk Development in Young Adults (Cardia) Study. *Control Clin Trials* **8**, 68S-73S (1987).

5. Redline S, Tishler PV, Schluchter M, Aylor J, Clark K, Graham G. Risk factors for sleep-disordered breathing in children. Associations with obesity, race, and respiratory problems. *Am J Respir Crit Care Med* **159**, 1527-1532 (1999).

6. Dean DA, 2nd*, et al.* Scaling Up Scientific Discovery in Sleep Medicine: The National Sleep Research Resource. *Sleep* **39**, 1151-1164 (2016).

7. Redline S*, et al.* The familial aggregation of obstructive sleep apnea. *Am J Respir Crit Care Med* **151**, 682-687 (1995).

8. Zhang GQ*, et al.* The National Sleep Research Resource: towards a sleep data commons. *J Am Med Inform Assoc* **25**, 1351-1358 (2018).

9. Fried LP*, et al.* The Cardiovascular Health Study: design and rationale. *Annals of epidemiology* **1**, 263-276 (1991).

10. O'Leary DH, Polak JF, Kronmal RA, Manolio TA, Burke GL, Wolfson SK, Jr. Carotid-artery intima and media thickness as a risk factor for myocardial infarction and stroke in older adults. Cardiovascular Health Study Collaborative Research Group. *The New England journal of medicine* **340**, 14-22 (1999).

11. O'Leary DH*, et al.* Use of sonography to evaluate carotid atherosclerosis in the elderly. The Cardiovascular Health Study. CHS Collaborative Research Group. *Stroke; a journal of cerebral circulation* **22**, 1155-1163 (1991).

12. Bowden DW*, et al.* Review of the Diabetes Heart Study (DHS) family of studies: a comprehensively examined sample for genetic and epidemiological studies of type 2 diabetes and its complications. *Rev Diabet Stud* **7**, 188-201 (2010).

13. Castelli WP. Epidemiology of coronary heart disease: the Framingham study. *Am J Med* **76**, 4-12 (1984).

14. Kannel WB, Castelli WP, Gordon T, McNamara PM. Serum cholesterol, lipoproteins, and the risk of coronary heart disease. The Framingham study. *Annals of internal medicine* **74**, 1-12 (1971).

15. Kannel WB, Dawber TR, Kagan A, Revotskie N, Stokes J, 3rd. Factors of risk in the development of coronary heart disease--six year follow-up experience. The Framingham Study. *Annals of internal medicine* **55**, 33-50 (1961).

16. Kannel WB, Feinleib M, McNamara PM, Garrison RJ, Castelli WP. An investigation of coronary heart disease in families. The Framingham offspring study. *American journal of epidemiology* **110**, 281-290 (1979).

17. Investigators F. Multi-center genetic study of hypertension: The Family Blood Pressure Program (FBPP). *Hypertension* **39**, 3-9 (2002).

18. Daniels PR*, et al.* Familial aggregation of hypertension treatment and control in the Genetic Epidemiology Network of Arteriopathy (GENOA) study. *Am J Med* **116**, 676-681 (2004).

19. Liu Y*, et al.* Pharmacogenetic association of the APOA1/C3/A4/A5 gene cluster and lipid responses to fenofibrate: the genetics of lipid-lowering drugs and diet network study. *Pharmacogenet Genomics* **19**, 161-169 (2009).

20. Higgins M*, et al.* NHLBI Family Heart Study: objectives and design. *American journal of epidemiology* **143**, 1219-1228 (1996).

21. Patsch JR*, et al.* Relation of triglyceride metabolism and coronary artery disease. Studies in the postprandial state. *Arteriosclerosis and thrombosis : a journal of vascular biology / American Heart Association* **12**, 1336-1345 (1992).

22. GenSalt Collaborative Research G. GenSalt: rationale, design, methods and baseline characteristics of study participants. *J Hum Hypertens* **21**, 639-646 (2007).

23. Kral BG*, et al.* A common variant in the CDKN2B gene on chromosome 9p21 protects against coronary artery disease in Americans of African ancestry. *J Hum Genet* **56**, 224-229 (2011).

24. Bray PF*, et al.* Heritability of platelet function in families with premature coronary artery disease. *J Thromb Haemost* **5**, 1617-1623 (2007).

25. Sorlie PD*, et al.* Design and implementation of the Hispanic Community Health Study/Study of Latinos. *Annals of epidemiology* **20**, 629-641 (2010).

26. Arnett DK*, et al.* Sibling correlation of left ventricular mass and geometry in hypertensive African Americans and whites: the HyperGEN study. Hypertension Genetic Epidemiology Network. *Am J Hypertens* **14**, 1226-1230 (2001).

27. Williams RR*, et al.* NHLBI family blood pressure program: methodology and recruitment in the HyperGEN network. Hypertension genetic epidemiology network. *Annals of epidemiology* **10**, 389-400 (2000).

28. Fuqua SR*, et al.* Recruiting African-American research participation in the Jackson Heart Study: methods, response rates, and sample description. *Ethnicity & disease* **15**, S6-18-29 (2005).

29. Taylor HA, Jr., *et al.* Toward resolution of cardiovascular health disparities in African Americans: design and methods of the Jackson Heart Study. *Ethnicity & disease* **15**, S6-4-17 (2005).

30. Wilson JG, *et al.* Study design for genetic analysis in the Jackson Heart Study. *Ethnicity & disease* **15**, S6-30-37 (2005).

31. Bild DE, *et al.* Multi-Ethnic Study of Atherosclerosis: objectives and design. *American journal of epidemiology* **156**, 871-881 (2002).

32. Ellinor PT, Low AF, Patton KK, Shea MA, Macrae CA. Discordant atrial natriuretic peptide and brain natriuretic peptide levels in lone atrial fibrillation. *Journal of the American College of Cardiology* **45**, 82-86 (2005).

33. Ellinor PT, Yoerger DM, Ruskin JN, MacRae CA. Familial aggregation in lone atrial fibrillation. *Human genetics* **118**, 179-184 (2005).

34. Mitchell BD, *et al.* Genetic and environmental contributions to cardiovascular risk factors in Mexican Americans. The San Antonio Family Heart Study. *Circulation* **94**, 2159-2170 (1996).

35. Hawley NL, *et al.* Prevalence of adiposity and associated cardiometabolic risk factors in the Samoan genome-wide association study. *Am J Hum Biol* **26**, 491-501 (2014).

36. Minster RL, *et al.* A thrifty variant in CREBRF strongly influences body mass index in Samoans. *Nat Genet* **48**, 1049-1054 (2016).

37. Ranade K, *et al.* A genome scan for hypertension susceptibility loci in populations of Chinese and Japanese origins. *Am J Hypertens* **16**, 158-162 (2003).

38. Wu KD, *et al.* Clustering and heritability of insulin resistance in Chinese and Japanese hypertensive families: a Stanford-Asian Pacific Program in Hypertension and Insulin Resistance sibling study. *Hypertens Res* **25**, 529-536 (2002).

39. Design of the Women's Health Initiative clinical trial and observational study. The Women's Health Initiative Study Group. *Control Clin Trials* **19**, 61-109 (1998).

40. Anderson GL, *et al.* Effects of conjugated equine estrogen in postmenopausal women with hysterectomy: the Women's Health Initiative randomized controlled trial. *JAMA : the journal of the American Medical Association* **291**, 1701-1712 (2004).

41. Manson JE, *et al.* Menopausal Hormone Therapy and Long-term All-Cause and Cause-Specific Mortality: The Women's Health Initiative Randomized Trials. *JAMA : the journal of the American Medical Association* **318**, 927-938 (2017).

42. Rao SS, *et al.* A 3D map of the human genome at kilobase resolution reveals principles of chromatin looping. *Cell* **159**, 1665-1680 (2014).
