# Supplementary material for: Chromosome Xq23 is associated with lower atherogenic lipid concentrations and favorable cardiometabolic indices
Source: Nat Commun. 2021 Apr 12;12:2182. doi: 10.1038/s41467-021-22339-1 (PMC8042019; doi:10.1038/s41467-021-22339-1)
Supplement: Supplementary file 3 — Reporting Summary [file 41467_2021_22339_MOESM3_ESM.pdf]

nature research

Corresponding author(s): Pradeep Natarajan, Gina Peloso

Last updated by author(s): Feb 9, 2021

Reporting Summary

Nature Research wishes to improve the reproducibility of the work that we publish. This form provides structure for consistency and transparency in reporting. For further information on Nature Research policies, see our Editorial Policies and the Editorial Policy Checklist.

Statistics

For all statistical analyses, confirm that the following items are present in the figure legend, table legend, main text, or Methods section.

n/a	Confirmed	
☐	☒	The exact sample size (*n*) for each experimental group/condition, given as a discrete number and unit of measurement
☐	☒	A statement on whether measurements were taken from distinct samples or whether the same sample was measured repeatedly
☐	☒	The statistical test(s) used AND whether they are one- or two-sided<br>*Only common tests should be described solely by name; describe more complex techniques in the Methods section.*
☐	☒	A description of all covariates tested
☐	☒	A description of any assumptions or corrections, such as tests of normality and adjustment for multiple comparisons
☐	☒	A full description of the statistical parameters including central tendency (e.g. means) or other basic estimates (e.g. regression coefficient) AND variation (e.g. standard deviation) or associated estimates of uncertainty (e.g. confidence intervals)
☐	☒	For null hypothesis testing, the test statistic (e.g. *F*, *t*, *r*) with confidence intervals, effect sizes, degrees of freedom and *P* value noted<br>*Give P values as exact values whenever suitable.*
☒	☐	For Bayesian analysis, information on the choice of priors and Markov chain Monte Carlo settings
☐	☒	For hierarchical and complex designs, identification of the appropriate level for tests and full reporting of outcomes
☐	☒	Estimates of effect sizes (e.g. Cohen's *d*, Pearson's *r*), indicating how they were calculated

Our web collection on statistics for biologists contains articles on many of the points above.

Software and code

Policy information about availability of computer code

Data collection	Data from contributing studies in the freeze 8 release of TOPMed was merged together and QC'ed using R v3.6.0. Results from the GTex portal V7 (https://gtexportal.org) were downloaded.
Data analysis	We used the ENCORE server implementing saige-0.29.4.4 (https://encore.sph.umich.edu), R v3.6.0, the GENESIS package v2.13.11 (https://www.bioconductor.org/packages/ release/bioc/vignettes/GENESIS/inst/doc/assoc_test.html), plink v1.9, and BOLT-LMM v2.3.1 to perform analyses.

For manuscripts utilizing custom algorithms or software that are central to the research but not yet described in published literature, software must be made available to editors and reviewers. We strongly encourage code deposition in a community repository (e.g. GitHub). See the Nature Research guidelines for submitting code & software for further information.

Data

Policy information about availability of data

All manuscripts must include a data availability statement. This statement should provide the following information, where applicable:

- Accession codes, unique identifiers, or web links for publicly available datasets
- A list of figures that have associated raw data
- A description of any restrictions on data availability

Source data are provided with this paper. Controlled access of the individual-level TOPMed data is available through dbGAP, and the individual-level UK Biobank data is available upon application to the UK Biobank. FinnGen summary-level data is fully freely available at https://www.finngen.fi/en/access_results. Individual-level access to FinnGen and HUNT cohorts may be obtained through reasonable request and suitable institutional review board approvals. The dbGaP accessions for TOPMed cohorts are as follows: Atherosclerosis Risk in Communities (ARIC) phs001211 [https://www.ncbi.nlm.nih.gov/projects/gap/cgi-bin/study.cgi?study_id=phs001211.v3.p2] and phs000280 [https://www.ncbi.nlm.nih.gov/projects/gap/cgi-bin/study.cgi?study_id=phs000280.v1.p1]; Old Order Amish

phs000956 [https://www.ncbi.nlm.nih.gov/projects/gap/cgi-bin/study.cgi?study_id=phs000956.v1.p1] and phs000391 [https://www.ncbi.nlm.nih.gov/projects/gap/cgi-bin/study.cgi?study_id=phs000391.v1.p1]; Mt Sinai BioMe Biobank phs001644 [https://www.ncbi.nlm.nih.gov/projects/gap/cgi-bin/study.cgi?study_id=phs001644.v1.p1] and phs000925 [https://www.ncbi.nlm.nih.gov/projects/gap/cgi-bin/study.cgi?study_id=phs000925.v1.p1]; Coronary Artery Risk Development in Young Adults (CARDIA) phs001612 [https://www.ncbi.nlm.nih.gov/projects/gap/cgi-bin/study.cgi?study_id=phs001612.v1.p1] and phs000285 [https://www.ncbi.nlm.nih.gov/projects/gap/cgi-bin/study.cgi?study_id=phs000285.v3.p2]; Cleveland Family Study (CFS) phs000954 [https://www.ncbi.nlm.nih.gov/projects/gap/cgi-bin/study.cgi?study_id=phs000954.v3.p2] and phs000284 [https://www.ncbi.nlm.nih.gov/projects/gap/cgi-bin/study.cgi?study_id=phs000284.v2.p1]; Cardiovascular Health Study (CHS) phs001368 [https://www.ncbi.nlm.nih.gov/projects/gap/cgi-bin/study.cgi?study_id=phs001368.v2.p2]; Diabetes Heart Study (DHS) phs001412 [https://www.ncbi.nlm.nih.gov/projects/gap/cgi-bin/study.cgi?study_id=phs001412.v2.p1] and phs001012 [https://www.ncbi.nlm.nih.gov/projects/gap/cgi-bin/study.cgi?study_id=phs001012.v1.p1]; Framingham Heart Study (FHS) phs000974 [https://www.ncbi.nlm.nih.gov/projects/gap/cgi-bin/study.cgi?study_id=phs000974.v1.p1] and phs000007 [https://www.ncbi.nlm.nih.gov/projects/gap/cgi-bin/study.cgi?study_id=phs000007.v32.p13]; Genetic Epidemiology Network of Arteriopathy (GENOA) phs001345 [https://www.ncbi.nlm.nih.gov/projects/gap/cgi-bin/study.cgi?study_id=phs001345.v2.p1] and phs001238 [https://www.ncbi.nlm.nih.gov/projects/gap/cgi-bin/study.cgi?study_id=phs001238.v1.p1]; Genetics of Lipid-Lowering Drugs and Diet Network (GOLDN) phs001359 [https://www.ncbi.nlm.nih.gov/projects/gap/cgi-bin/study.cgi?study_id=phs001359.v2.p1] and phs000741 [https://www.ncbi.nlm.nih.gov/projects/gap/cgi-bin/study.cgi?study_id=phs000741.v2.p1]; Genetic Epidemiology Network of Salt Sensitivity (GenSalt) phs001217 [https://www.ncbi.nlm.nih.gov/projects/gap/cgi-bin/study.cgi?study_id=phs001217.v2.p1] and phs000784 [https://www.ncbi.nlm.nih.gov/projects/gap/cgi-bin/study.cgi?study_id=phs000784.v1.p1]; Genetic Studies of Atherosclerosis Risk (GeneSTAR) phs001218 [https://www.ncbi.nlm.nih.gov/projects/gap/cgi-bin/study.cgi?study_id=phs001218.v2.p1] and phs000375 [https://www.ncbi.nlm.nih.gov/projects/gap/cgi-bin/study.cgi?study_id=phs000375.v1.p1]; Hispanic Community Health Study – Study of Latinos (HCHS/SOL) phs001395 [https://www.ncbi.nlm.nih.gov/projects/gap/cgi-bin/study.cgi?study_id=phs001395.v1.p1] and phs000810 [https://www.ncbi.nlm.nih.gov/projects/gap/cgi-bin/study.cgi?study_id=phs000810.v1.p1]; Hypertension Genetic Epidemiology Network and Genetic Epidemiology Network of Arteriopathy (HyperGEN) phs001293 [https://www.ncbi.nlm.nih.gov/projects/gap/cgi-bin/study.cgi?study_id=phs001293.v2.p1]; Jackson Heart Study (JHS) phs000964 [https://www.ncbi.nlm.nih.gov/projects/gap/cgi-bin/study.cgi?study_id=phs000964.v1.p1] and phs000286 [https://www.ncbi.nlm.nih.gov/projects/gap/cgi-bin/study.cgi?study_id=phs000286.v4.p1]; Multi-Ethnic Study of Atherosclerosis (MESA) phs001416 [https://www.ncbi.nlm.nih.gov/projects/gap/cgi-bin/study.cgi?study_id=phs001416.v1.p1] and phs000209 [https://www.ncbi.nlm.nih.gov/projects/gap/cgi-bin/study.cgi?study_id=phs000209.v7.p2]; Massachusetts General Hospital Atrial Fibrillation Study (MGH_AF) phs001062 [https://www.ncbi.nlm.nih.gov/projects/gap/cgi-bin/study.cgi?study_id=phs001062.v4.p2] and phs001001 [https://www.ncbi.nlm.nih.gov/projects/gap/cgi-bin/study.cgi?study_id=phs001001.v1.p1]; San Antonio Family Study (SAFS) phs001215 [https://www.ncbi.nlm.nih.gov/projects/gap/cgi-bin/study.cgi?study_id=phs001215.v3.p2] and phs000462 [https://www.ncbi.nlm.nih.gov/projects/gap/cgi-bin/study.cgi?study_id=phs000462.v1.p1]; Samoan Adiposity Study phs000972 [https://www.ncbi.nlm.nih.gov/projects/gap/cgi-bin/study.cgi?study_id=phs000972.v1.p1] and phs000914 [https://www.ncbi.nlm.nih.gov/projects/gap/cgi-bin/study.cgi?study_id=phs000914.v1.p1]; Taiwan Study of Hypertension using Rare Variants (THRV) phs001387 [https://www.ncbi.nlm.nih.gov/projects/gap/cgi-bin/study.cgi?study_id=phs001387.v2.p1]; Women's Health Initiative (WHI) phs001237 [https://www.ncbi.nlm.nih.gov/projects/gap/cgi-bin/study.cgi?study_id=phs001237.v2.p1] and phs000200 [https://www.ncbi.nlm.nih.gov/projects/gap/cgi-bin/study.cgi?study_id=phs000200.v12.p3].

Field-specific reporting

Please select the one below that is the best fit for your research. If you are not sure, read the appropriate sections before making your selection.

☒ Life sciences ☐ Behavioural & social sciences ☐ Ecological, evolutionary & environmental sciences

For a reference copy of the document with all sections, see nature.com/documents/nr-reporting-summary-flat.pdf

Life sciences study design

All studies must disclose on these points even when the disclosure is negative.

Sample size	Individuals with TOPMed whole genome sequencing available and blood lipid levels were included in the study. This is the largest study of the X chromosome with WGS data.
Data exclusions	Individuals were excluded when their genotype determine sex did not match phenotype reported sex (n=6) and individuals < 18 years old where excluded (n=865). Our exclusion criteria was pre-established. We excluded individuals that had discordant sex since our focus was on the X chromosome and excluded individuals < 18 years old as lipid levels in children have a different distribution than in adults.
Replication	We sought replication of our primary analysis in additional samples (HUNT, UKBB, and FINGEN). All replication samples were independently collected and only the studies listed were attempted for replication. All replication attempts were successful.
Randomization	No randomization was performed. Since this is a population based study and did not focus on a treatment effect, randomization was not performed.
Blinding	No blinding was performed. Blinding was not performed as there was no randomization. Investigators did not have any access to identifying information.

Reporting for specific materials, systems and methods

We require information from authors about some types of materials, experimental systems and methods used in many studies. Here, indicate whether each material, system or method listed is relevant to your study. If you are not sure if a list item applies to your research, read the appropriate section before selecting a response.

Materials & experimental systems

n/a	Involved in the study
☒	☐ Antibodies
☒	☐ Eukaryotic cell lines
☒	☐ Palaeontology and archaeology
☒	☐ Animals and other organisms
☐	☒ Human research participants
☒	☐ Clinical data
☒	☐ Dual use research of concern

Methods

n/a	Involved in the study
☒	☐ ChIP-seq
☒	☐ Flow cytometry
☒	☐ MRI-based neuroimaging

Human research participants

Policy information about studies involving human research participants

Population characteristics	Baseline characteristics of TOPMed participants in discovery genetic association analyses by cohort can be found in Supplementary Table 1. Characteristics of the replication cohorts are available in Supplementary Tables 6, 7, and 12
Recruitment	Participant recruitment varied based on the study the participant was in. Details on recruitment criteria for each study is available in the supplemental material.
Ethics oversight	Individual studies in TOPMed approved each studies data collection. Study participants provided consent per each study's IRB approved protocol. These data were secondarily analyzed through a protocol approved by the Partners Healthcare IRB and Boston University IRB.<br>The FinnGen project is approved by Finnish Institute for Health and Welfare (THL).<br>HUNT was approved by the Data Inspectorate and the Regional Ethics Committee for Medical Research in Norway (REK: 2014/144). All HUNT participants gave informed consent.

Note that full information on the approval of the study protocol must also be provided in the manuscript.
